# The Gambian epauletted fruit bat shows increased genetic divergence in the Ethiopian highlands and in an area of rapid urbanization

**DOI:** 10.1002/ece3.4709

**Published:** 2018-12-11

**Authors:** Silke A. Riesle‐Sbarbaro, Kofi Amponsah‐Mensah, Stefan de Vries, Violaine Nicolas, Aude Lalis, Richard Suu‐Ire, Andrew A. Cunningham, James L. N. Wood, David R. Sargan

**Affiliations:** ^1^ Department of Veterinary Medicine University of Cambridge Cambridge UK; ^2^ Institute of Zoology Zoological Society of London, Regents Park London UK; ^3^ Institut Systématique Evolution Biodiversité (ISYEB) Sorbonne Université, MNHN, CNRS, EPHE Paris France; ^4^ Centre for African Wetlands University of Ghana Legon, Accra Ghana; ^5^ Wildlife Division of the Forestry Commission Accra Ghana; ^6^ Present address: Poultry Research and Development MSD Animal Health Boxmeer The Netherlands

**Keywords:** Africa, bottleneck, *Epomophorus gambianus*, gene flow, mitochondrial DNA, nuclear microsatellites

## Abstract

The Gambian epauletted fruit bat (*Epomophorus gambianus*) is an abundant species that roosts in both urban and rural settings. The possible role of *E. gambianus* as a reservoir host of zoonotic diseases underlines the need to better understand the species movement patterns. So far, neither observational nor phylogenetic studies have identified the dispersal range or behavior of this species. Comparative analyses of mitochondrial and nuclear markers from 20 localities across the known distribution of *E. gambianus* showed population panmixia, except for the populations in Ethiopia and southern Ghana (Accra and Ve‐Golokwati). The Ethiopian population may be ancestral and is highly divergent to the species across the rest of its range, possibly reflecting isolation of an ancient colonization along an east–west axis. Mitochondrial haplotypes in the Accra population display a strong signature of a past bottleneck event; evidence of either an ancient or recent bottleneck using microsatellite data, however, was not detected. Demographic analyses identified population expansion in most of the colonies, except in the female line of descent in the Accra population. The molecular analyses of the colonies from Ethiopia and southern Ghana show gender dispersal bias, with the mitochondrial DNA fixation values over ten times those of the nuclear markers. These findings indicate free mixing of the species across great distances, which should inform future epidemiological studies.

## 1 INTRODUCTION

Migration and dispersal are important behaviors that drive evolution in many animal populations. In broad terms, migration is a seasonal two‐way movement usually associated with breeding sites, whereas dispersal is a one‐way movement often undertaken by sexually immature individuals (Moussy et al., 2013). Both types of movement can shape the genetic structure of populations via gene flow, which leads to a reduction in genetic variation between dispersed populations. Gene flow in bat species can be greatly facilitated by the ability to perform true flight, with some bat populations showing panmixia, or near panmixia, across much of their geographical distribution (Chen et al., 2010; Moussy et al., 2015; Peel et al., 2013; Petit & Mayer, 1999; Russell, Medellin, & McCracken, 2005; Webb & Tidemann, 1996). However, these movements can also be markedly restricted (Entwistle, Racey, & Speakman, 2000; Rossiter, Jones, Ransome, & Barratt, 2000) or gender‐biased due to philopatric behaviors, which are reflected as different degrees of genetic structuring. For example, sex‐biased dispersal in bat species is mainly due to female philopatry (Rossiter, Jones, Ransome, & Barratt, 2002; Rydell, 1989). The opposite behavior has also been documented, that is, longer distances of female bat dispersal compared with males (Nagy, Gunther, Knornschild, & Mayer, 2013; Nagy, Heckel, Voigt, & Mayer, 2007).

Long‐distance animal movements also can drive the transmission of pathogens within and between species, shaping epidemiological dynamics among wildlife populations. For example, *Eidolon helvum*, the most populous large fruit bat in sub‐Saharan Africa that is often found in urban areas including megacities (DeFrees & Wilson, 1988; Hayman, McCrea, et al., 2012), has the largest panmictic population among terrestrial mammals, showing similar seroprevalences against henipaviruses and Lagos bat virus among disparate continental African countries (Peel et al., 2013). Animal dispersal may have important implications for public health, but the true role that these movement patterns play in pathogen transmission is still not well understood (Suzán et al., 2015). For example, although there is a generalized assumption that migratory animals increase pathogen dispersal (Figuerola & Green, 2000; Rappole, Derrickson, & Hubalek, 2000; Reed, Meece, Henkel, & Shukla, 2003), it has been suggested that in some circumstances, the opposite can be true, for example, migration can allow healthy hosts to escape infected habitats, reducing the impact of disease on a population (Altizer, Bartel, & Han, 2011; Hall, Altizer, & Bartel, 2014). This highlights the need for accurate data and a better understanding of animal movement, particularly for potential reservoir species of zoonotic diseases.

**Figure 1:**
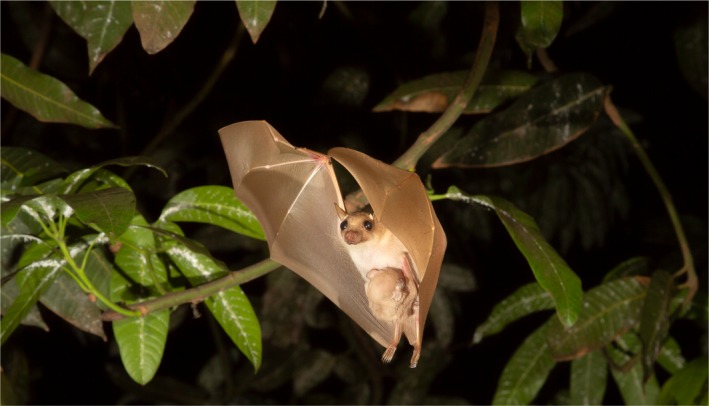
Picture of a female specimen of *Epomophorus gambianus *flying into a mango tree, with a pup attached. Photograph was taken in Greater Accra, year 2015

**Figure 2:**
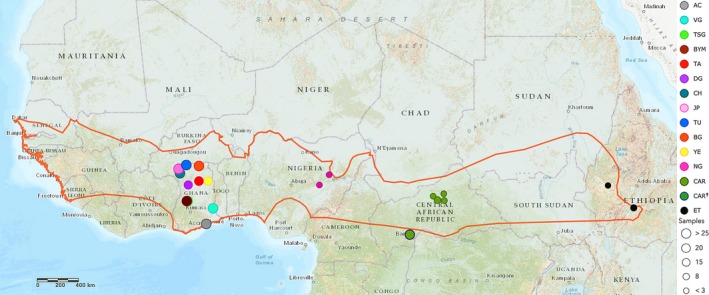
Map showing *E. gambianus* geographical distribution (extracted from IUCN and shown with an orange line) and sampling sites (circles). Bubble size reflects sample size. The legend shows the color‐coded sampling sites, where AC: Greater Accra, VG: Ve‐Golokwati, TSG: Tanoboase Sacred Grove, BYM: Buoyem, TA: Tamale, DG: Damongo, CH: Charia, JP: Jirapa, TU: Tumu, BG: Bolgatanga, YE: Yendi, CAR: Central African Republic, NG: Nigeria, ET: Ethiopia, CAR**^†^**: CYTB sequences downloaded from GenBank

*Epomophorus gambianus* (Figure 1), commonly known as the Gambian epauletted fruit bat, is a potential reservoir host of Ebola virus (Hayman, Yu, et al., 2012). Across its distribution (Figure 2), *E. gambianus* has been reported to roost in small colonies of up to 100 individuals (Boulay & Robbins, 1989). It is described as a lowland species usually found below 500 meters above sea level (m a.s.l.), apart from in Ethiopia, where it has been reported to occur up to nearly 2,000 m a.s.l. (Mickelburgh, Hutson, & Bergmans, 2008). *Epomophorus gambianus* is a medium‐sized bat that has not been described previously as undergoing migration or long‐distance dispersal (Boulay & Robbins, 1989; Mickelburgh et al., 2008). Bats which fly long distances have morphological characteristics (ecomorphology) that enable energy‐efficient flight, such as a high aspect ratio (long, narrow wings), which favors aerodynamic efficiency and lower losses of energy in flight, and high wing loading (low wing area relative to body mass), which correlates with high speed flights but low maneuverability (Norberg & Rayner, 1987; Olival, 2012). Norberg and Rayner (1987) determined that *E. gambianus* has the characteristics of a fast, maneuverable and agile flyer (e.g., low aspect ratio, relatively short wingspan, high wing loading, and an average wingtip shape) that are not typical features for long‐distance flight.

Population genetics has been increasingly used to elucidate wildlife movement, particularly for species that are difficult to track directly. Mitochondrial DNA (mtDNA) has historically been selected as a molecular marker for phylogeographic studies and has also been widely used for the study of speciation (Boattini et al., 2013; Song, Lan, & Kohn, 2014; Talbot, Vonhof, Broders, Fenton, & Keyghobadi, 2016). For example, sequencing of the mitochondrial cytochrome b gene (CYTB) revealed a polyphyletic relationship between *E. gambianus* *and* *Micropteropus pusillus* (species within the Epomophorini tribe). Using only a region of the mtDNA, however, did not robustly identify introgression between these species (Nesi, Nakoune, Cruaud, & Hassanin, 2011). To assess this, either complete mitochondrial genomes can be used (Riesle‐Sbarbaro et al., 2016) or the analysis can be complemented using biparentally inherited markers such as microsatellites. Microsatellites are nuclear DNA (ncDNA) markers commonly used in phylogeographic studies of populations (Goldstein & Pollock, 1997; Hindley, Graham, Pulgarin, & Burg, 2018; Muriira, Muchugi, Yu, Xu, & Liu, 2018; Rossiter, Benda, Dietz, Zhang, & Jones, 2007). So far, the population‐based phylogeography of *E. gambianus* has not been investigated. In this study, we aim to determine the genetic structure of this species across its range using both nuclear and mitochondrial markers, not only to increase the currently limited knowledge of the ecology and demographic history of this bat, but also to inform future epidemiological studies, by answering the following questions:

1. Is *E. gambianus* freely mixing across its entire geographic distribution? If not, is there a pattern of isolation by distance or complete gene flow disruption?
2. What can be concluded about the demographic history of *E. gambianus*?

## 2 MATERIALS AND METHODS

### 2.1 Sample collection

Tissue samples or extracted DNA of 308 *E. gambianus* was collected from 20 localities from across the species geographical distribution (IUCN, 2016) along a linear east–west axis (Figure 2; Table 1). Eleven colonies were sampled within Ghana, 2013–2015, by the collection of 3‐ to 4‐mm‐diameter wing membrane biopsies of using a biopsy punch (Henry Schein, UK), while tissue samples from Nigeria and Ethiopia and extracted DNA samples from the Central African Republic were acquired from museum specimens (Table 1).

**Table 1:** Sampling details and number of *E. gambianus *genetic markers included in the analyses

Site ID	LOCATION	Longitude		Latitude	Number of samples typed			Date	Source
					CYTB	D‐loop	SSR		
AC	Ghana, Greater Accra	0°11'03.2″W		5°35'13.6″N	25	25	25	2015	SRS
VG	Ghana, Ve‐Golokwati	0°26'16.1″E		6°59'46.0″N	25	25	25	2015	SRS
TSG	Ghana, Tanoboase	1°51'25.1″W		7°39'55.5″N	25	25	24	2015	SRS
BYM	Ghana, Buoyem	1°57'00.0″W		7°40'00.0″N	47	25	27	2015	SRS
TA	Ghana, Tamale	0°50'36.1″W		9°25'33.8″N	20	20	20	2015	SRS
DG	Ghana, Damongo	1°48'45.4″W		9°05'07.8″N	17	17	17	2015	SRS
CH	Ghana, Charia	2°34'34.0″W		10°06'46.6″N	25	25	25	2015	SRS
JP	Ghana, Jirapa	2°42'01.3″W		10°31'59.1″N	25	25	25	2015	SRS
TU	Ghana, Tumu	1°59'16.2″W		10°52'27.7″N	24	25	25	2015	SRS
BG	Ghana, Bolgatanga	0°51'25.7″W		10°47'48.4″N	25	25	25	2015	SRS
YE	Ghana, Yendi	0°00'24.1″W		9°26'22.5″N	18	18	18	2015	SRS
GO	Nigeria, Gombe	10°57'8.5″E		9°59'12.4″N	3	3	2	1997	SFM
PL	Nigeria, Plateau	10°3'27.0″E		9°4'39.9″N	3	3	1	1997	SFM
SA	CAR, Sangba	20°8'24.9″E		7°48'26.1″N	9	8	5	1998	MNHN
BH	CAR, Bohou	22°2'51.5″E		6°44'20.6″N	4	3	3	1998	MNHN
KO	CAR, Koumbala	20°54'10.7″E		8°45'38.5″N	2	2	2	1998	MNHN
BA	CAR, Bangoran	20°20'35.3″E		8°4'55.6″N	2	2	2	1998	MNHN
BNE	CAR, Bangui^a^	18°35'04.5″E		4°22'07.3″N	22	–	–	2008	Nesi et al. (2011)
SI	Ethiopia, Sidama	38°27'57.1″E		7°1'45.7″N	6	6	7	1973	SFM
DI	Ethiopia, Didessa	36°9'7.7″E		9°1'51.6″N	3	3	–	1975	SFM
**GH**	**Ghana **(AC + VG + TS + BYM + TA + DG + CH + JP + TU + BG + YE)				**254**	**255**	**256**		
**NG**	**Nigeria (**GO + PL)				**6**	**6**	**3**		
**CAR**	**CAR (**SA + BH + KO + BA ± BNE)				**39**	**15**	**11**		
**ET**	**Ethiopia (**SI + DI)				**9**	**9**	**7**		
**WS**	**West **(GH + NG)				**260**	**261**	**259**		
**CT**	**Central **(GH + NG + CAR)				**299**	**276**	**270**		
**EG**	**Total *E. gambianus ***(All locations)				**308**	**285**	**277**		
OG	**Out‐group**		*Epomops * *franqueti*		19	21	–	2012	MNHN
OG1	**Out‐group**		*Rousettus aegyptiacus*		1	1	–		GenBank^b^ AB205183

Notes

Sampling sites are color coded in Figure 2. Year of sample collection is included (Date), and source of samples are noted as: SRS (first author), SFM (Senckenberg Forschungsinstitut Mammalogie), and MNHN (Museum National d'Histoire Naturelle, Paris).

a CYTB sequences downloaded from GenBank. Accession number included in Nesi et al. (2011).

b Mitochondrial DNA sequences downloaded from GenBank. Accession number included in Source.

### 2.2 Sequencing and genotyping

Genomic DNA was extracted using the DNeasy Blood and Tissue Kit (Qiagen Ltd., UK). Samples obtained from museum collections were extracted using the QIAamp DNA FFPE Tissue Kit (Qiagen Ltd., UK). Tissues sampled from the pectoral muscle were digested overnight (24 hr) using 180 μl of ATL buffer and 40 μl of proteinase K. A paired‐end Illumina sequencing library was constructed, as previously described (Riesle‐Sbarbaro et al., 2016), and the mitochondrial genome of *E. gambianus *was assembled and annotated. Primers for CYTB and D‐loop regions were selected (see Supporting information, Table S1) for Sanger sequencing, and DNA was amplified in 10 μl of reaction mix containing 2 ng of template DNA, 10 μM of forward and reverse primers, and 5 μl of MegaMix‐Gold master mix. Touchdown PCR settings used to amplify the CYTB fragments were as follows: 5 min at 95**°**C; followed by 12 cycles of 20 s at 94**°**C, 20 s at 66**°**C (decreasing one degree per cycle), and 20 s at 72**°**C; 30 cycles of 1 min at 94**°**C, 1 min at 54**°**C, and 1 min at 72**°**C; and a final extension of 7 min at 72**°**C; and for D‐loop, the conditions used were as follows: 5 min at 95**°**C; 40 cycles of 1 min at 93**°**C, 90 s at 55**°**C and 2 min at 72**°**C; and a final extension of 7 min at 72**°**C. PCR products were screened using 2% agarose gel electrophoresis and purified using the Exonuclease I/Shrimp Alkaline Phosphatase Method (ExoSAP‐IT kit, Affymetrix Ltd., UK). PCR products were Sanger sequenced externally (Source Bioscience, UK, Ltd.), and Geneious v8.1 software was used for quality‐trimming and alignment of the sequences. Both CYTB and D‐loop sequences were realigned using the software Gblocks 0.91b (Castresana, 2000) under semistringent parameters. CYTB sequences were kept in two conserved alignment blocks retaining 99% of the original alignment (532 bp). Due to the high variability of D‐loop sequences, some PCRs failed to generate unique products, so four alignment blocks were generated using only 75% of the original sequence information (414 bp).

Microsatellite primers for nuclear DNA (ncDNA) were developed using *E. gambianus* paired‐end reads (that passed the Illumina filters) imported to the “SSR_Pipeline” (Miller, Knaus, Mullins, & Haig, 2013), which was programmed to identify simple microsatellite markers that presented at least 9 repeats of dinucleotide motifs or at least 5 repeats for tetranucleotide motifs (Supporting information, Table S2). From this output, an initial selection of 34 nuclear microsatellites was filtered to 20, according to the reproducibility of scoring and heterozygosity detected in 20 bats. Forward primers were extended with universal primers M13, T7, SP6, and T3 to their 5′ end to indirectly label them with fluorescent dyes (NED, 6‐FAM, VIC, and PET, respectively). PCRs (total of 15 μl mix) were amplified using the QIAGEN multiplex PCR kit (Qiagen Ltd., UK) with the following settings: 15 min at 95**°**C; 30 cycles of 30 s at 94**°**C, 90 s at 60**°**C, 60 s at 72**°**C; and an extension of 30 min at 60**°**C. Genotyping was outsourced (Source Bioscience, UK, Ltd.), and peaks were analyzed using the Geneious version 8.1 software.

### 2.3 Characterization of molecular markers

Samples from 308 bats were initially obtained for genotyping, but due to poor quality or low yield of DNA (reflected as ambiguous peaks or no amplification of SSR), 277 bats (144 females and 133 males) were included in subsequent analyses. The statistical power of both mtDNA and microsatellite markers in the dataset to detect significant population differentiation was tested using the software POWSIM (Ryman & Palm, 2006). A thousand simulations were run using the empirical values of the ncDNA dataset, detecting *F*_ST_ values from 0.001 to 0.01. To test the CYTB sequences, the dataset was adjusted for the organelle data (mtDNA) halving sample size (Larsson, Charlier, Laikre, & Ryman, 2008).

Null alleles and large allele dropout were checked using the software MICRO‐CHECKER (Van Oosterhout, Hutchinson, Wills, & Shipley, 2004). Each locus was tested for heterozygosity and departures of Hardy–Weinberg equilibrium (HWE) using the Adegenet package 2.0.0 (Jombart, 2008), correcting significance for multiple comparisons using the false discovery rate method (FDR) with the *p.adjust* function in R version 3.1.2 (R_Core_Team, 2014). HWE was tested with both the classical chi‐square test (based on the expected genotype frequencies) and an exact test based on Markov chain Monte Carlo (MCMC) permutations of alleles after 10,000 repetitions. Microsatellites that departed from HWE (L25, L26, and L36) or had a large proportion of missing peak calls (L23 and L15) were excluded from the analyses (Supporting information, Table S2), and HWE equilibrium of the colonies was checked afterward. Linkage disequilibrium (LD) was tested using the R package Adegenet and the software GENEPOP version 4.2 (Rousset, 2008). LD was tested using MCMC (100 replicates, 10,000 dememorization steps, and 10,000 randomizations). An additional verification of these results was performed using the software FSTAT version 2.9.3.2 (Goudet, 1995) and GenoDive version 2.0b27 (Meirmans & Van Tienderen, 2004).

### 2.4 Genetic diversity and demographic statistics

After selection of the ncDNA markers, descriptive statistics of each population and groups of populations were acquired by calculating the expected heterozygosity (*H*_E_), observed heterozygosity (*H*_o_), and corrected heterozygosity for unknown alleles (*H'*_T_) using both GenoDive software and the Adegenet package in R. The software packages BOTTLENECK version 1.2.02 (Piry, Luikart, & Cornuet, 1999) and M_P_Val (Garza & Williamson, 2001) were used to detect recent and past effective population size reduction from the ncDNA allele data of 17 loci. The BOTTLENECK analyses were calculated using as input values 12% of variance and 95% of single‐step mutations, as suggested by Piry et al. (1999), for 5,000 replications. The M ratio analyses, which calculate the mean ratio of allele size per locus to the range of alleles sizes and compare it with simulated values excepted under equilibrium (significance assumed as <5% of the M ratios generated below the observed value), were calculated assuming 90% of stepwise mutations and a combination of increasing values for ancestral theta = *θ* (1–10) and Δ*g* (2.5 and 3.5). We also compared the empirical M value to the critical M (Mc) using *θ* =10 and Δ*g* *=* *3.5* for 10,000 simulations of stable populations in the package Critical_M, which determines a past bottleneck when values lower than the Mc are detected. A graphical test of the distribution of alleles was analyzed as another approach to test a bottleneck event (Luikart, Allendorf, Cornuet, & Sherwin, 1998). In doing so, alleles of 17 loci were grouped into 10 allele frequency classes and then plotted as a histogram using R version 3.1.2.

Indices of genetic diversity and demographic parameters based on mitochondrial DNA were obtained using the CYTB marker (due to its greater quality). Calculation of genetic indices (number of haplotypes [*h*], haplotype diversity [*Hd*], nucleotide diversity [*π*], mean number of pairwise differences [*k*], number of polymorphic sites [*S*] and molecular diversity estimated from the number of polymorphic sites [*θ_s_*], and Class I and II neutrality statistics [*Tajima's* *D,* *Fu* *and* *Li's* D* *and* F*, Ramos‐Onsins and Rozas’ *R_2_* *and* *Fu's* *F*]) was computed using the software DNAsp v5.10 (Rozas & Rozas, 1995). *p* values and 95% confidence intervals were obtained by coalescence simulation over 5,000 replications. Haplotypic richness (*HR*), standardized to a minimum sample size (6 bats), was calculated using a rarefaction curve using the software Contrib (Petit, Mousadik, & Pons, 2008). Class III statistics (mismatch distributions) were analyzed for both spatial and demographic expansion models using ARLEQUIN version 3.5 (Excoffier, Laval, & Schneider, 2005). The demographic expansion model did not converge for the AC, YE, and ET populations; therefore, these indices and statistics are not shown for any colony. Bayesian skyline, constant size, and extended Bayesian skyline models (EBSP) were implemented in BEAST 2.4.8 software (Bouckaert et al., 2014; Heled & Drummond, 2008) to infer demographic history. For this, we used a substitution model HKY, selected in jModelTest version 2.1.7 (Posada, 2008), using strict linked and unlinked clock models with the concatenated (CYTB and D‐loop) mtDNA fragment. The MCMC was run twice for 30 million generations, sampling every 10,000 generations, discarding 10% as burn‐in and combined using the package LogCombiner 2.4.8. We compared models using TRACER 1.7.1 (Rambaut, Drummond, Xie, Baele, & Suchard, 2018) and selected the ESBP linked model as a better fit. The plot was done in R.

### 2.5 Phylogenetic analyses

A best nucleotide substitution model for the CYTB and concatenated mtDNA alignments was calculated with the Akaike information criterion (AIC) and the Bayesian information criterion in jModelTest. The model selected for the CYTB alignment was HKY + G, and for the concatenated fragment, the model selected was HKY + I + G. A gamma mixed model was also used for the latter. In order to quantify divergence times between the clades of *E. gambianus*, a CYTB substitution clock of 3%/My was initially used in Mega5 software (Hulva, Horacek, Strelkov, & Benda, 2004; Nabholz, Glemin, & Galtier, 2008; Tamura et al., 2011). The resulting divergence time, presented as millions of years ago (Mya), was checked and further calibrated against the best estimates of divergence time between the out‐group species (*Rousettus aegyptiacus *and *Epomops franqueti*) and *E. gambianus* (Hedges, Dudley, & Kumar, 2006; Hedges, Marin, Suleski, Paymer, & Kumar, 2015). Haplotype alignments were generated using the software DNAsp v5.10 for the processed fragments. Graphical representations of the interspecific relationships of the individuals were generated using tree‐like phylogenies and haplotype networks. Phylogenetic trees were generated using both maximum likelihood in PhyML version 3.0 (Guindon et al., 2010) and Bayesian inference in MrBayes version v3.2.5 (Ronquist & Huelsenbeck, 2003). Maximum‐likelihood trees (Supporting information, Figure S3B–D) were run with 1,000 bootstrap supports, and the Bayesian models were run with 6 simultaneous chains, sampled every 100 generations for 10^9^ generations, or until the standard deviation split frequencies reach 0.01. The first 25% of the trees were discarded. The output files were processed with FIGTREE (https://tree.bio.ed.ac.uk/software/figtree/). The haplotype networks were constructed with the software NETWORK version 5.0 (www.fluxus-engineering.com) using a Median‐Joining algorithm (Bandelt, Forster, & Röhl, 1999). To decrease the complexity of the reticulations in the graph, mutations at a given nucleotide were weighted according to their frequency (Supporting information, Table S4), increasing number of mutations on a particular position were deemed less informative and down‐weighted. Transversion changes were given three times the weight of transitions as the latter events are over 15 times more likely to occur in mammal mitochondria (Šnábel, 2012).

### 2.6 Population structure

Gene flow disruption, evidenced as population structure, was assessed using pairwise *F*‐statistics, hierarchical differentiation of the populations, and spatial‐genetic distance correlations. Pairwise differentiation between populations was tested for both molecular analogues, using *F*_ST_ and Φ_ST_ statistics (Weir & Cockerham, 1984). Pairwise exact tests were performed with 10,000 steps in the Markov chain, 10,000 dememorization steps, and 10,000 randomizations in the permutations test. Corrections were made for multiple comparisons using the FDR method. Specific statistics developed for multiallelic data (*G*_ST_, *Nei's G'*_ST_, *Hedrick's* *G'*_ST_ and *Jost's D*) were calculated using the GenoDive software. Hierarchical structure between groups of populations was also explored using molecular variance analyses (AMOVA) implemented in the software ARLEQUIN version 3.5 (Excoffier et al., 2005) under the same conditions. Isolation by distance (IBD) between populations was explored, for both mitochondrial and nuclear markers, by correlating the logarithmic geographical distances between colonies (kilometers) against Slatkin's linearized *F*_ST_ and Φ*_ST_*. Statistical significance was tested using a Mantel test with 10,000 permutations in both ARLEQUIN and R software.

To demonstrate population structure using ncDNA, a Bayesian clustering method was implemented using the complete dataset (277 bats, 15 loci) and the software STRUCTURE version 2.3.2 (Pritchard, Stephens, & Donnelly, 2000). Due to prior knowledge of the species ecology and expected migratory patterns, an *Admixture* model, with correlated allele frequencies (Falush, Stephens, & Pritchard, 2003), was selected. Sampling sites were used as priors (LOCPRIOR) to increase the power of the analyses (Hubisz, Falush, Stephens, & Pritchard, 2009), carrying out MCMC runs using 500,000 iterations as burn‐in, followed by 1,000,000 iterations. Sixteen groups were assigned (*K* = 1 to *K* = 16) for each run, and each *K* analysis was replicated 15 times. Because STRUCTURE is sensitive to sample size differences, a second model was explored decreasing the number of samples from AC and VG to an averaged number of 16 individuals (to balance the small sample size of ET). The numbers of bats included from the other colonies were also reduced, but only to 20 individuals due to the previous model result showing low structure signal. The software STRUCTURE HARVESTER web v0.6.94 (Earl & vonHoldt, 2011) was used to parse and format the replicated analyses. The best *K* that fitted the data based on the Evanno method (Evanno, Regnaut, & Goudet, 2005) was also explored with this software and verified using a discriminant analysis of principal components (DAPC, Jombart, Devillard, & Balloux, 2010) in the Adegenet package in R. The pipeline CLUMPAK version 1.1 (Kopelman, Mayzel, Jakobsson, Rosenberg, & Mayrose, 2015) was implemented, using a *LargeGreedy *algorithm, to align the samples of each *K* repetition and to graphically visualize it.

## 3 RESULTS

### 3.1 Indices of genetic diversity

From the total number of CYTB sequences (*n* = 308) included in the analysis, 87 unique haplotypes (*h*) were found (Table 2). The haplotype diversity (*Hd*) of the entire sample set of *E. gambianus* (EG) was high (0.91). Within Ghanaian colonies, the *Hd* was high overall (*Hd* over 0.87), but there was reduced *Hd* in the colonies sampled in Ve‐Golokwati (VG) town from the Volta region (*Hd *= 0.78) and particularly in Greater Accra (AC, *Hd *= 0.38). The bats sampled in AC presented the lowest haplotype diversity found in any of the colonies sampled. Outside Ghana (GH), both Nigerian (NG) and Ethiopian (ET) colonies presented lower *Hd *than average (0.60 and 0.72, respectively); however, these results are less robust due to having smaller sample sizes and collection from two separate colonies. In order to buffer the effect of uneven sample size between countries, the haplotype richness standardized to the minimum sample size (HR) was calculated. Although this standardized the results, AC maintained a particularly low diversity level (HR = 0.85) compared to the average (HR = 3.47).

**Table 2:** *Epomophorus gambianus* genetic diversity and demographic statistics within colonies (Population level) and by regional grouping (Group level)

	Mitochondrial DNA markers–CYTB														Nuclear DNA–microsatellites			
	Genetic indices						Class I					Class II	Class III		Nuclear diversity			
	Pop	*n*	*h*	*Hd*	±*SD*	*HR*	*D* *_T_*	D*	F*	*R*_2_	*S/d*	*Fu's F*	*rg*	MM	*n*	*A*	*A*_R_	*H*_o_
Population level	AC	25	2	0.38	0.09	0.85	1.25	1.16	1.38	0.19	2.63	5.54	0.673	B	25	10.3	6.02	0.83
	VG	25	13	0.78	0.09	3.06	−0.88	−1.45	−1.49	0.09	5.00	−3.83	0.063	U	25	10.6	6.67	0.84
	TSG	25	15	0.87	0.06	3.70	**−1.77**	**−2.60**	**−2.74**	***0.06***	7.24	**−6.46**	0.020	U	24	11.0	7.18	0.87
	BYM	25	16	0.93	0.04	4.09	−1.17	**−2.01**	**−2.05**	0.07	5.57	**−7.89**	0.043	U	27	10.8	7.38	0.83
	TA	20	12	0.87	0.07	3.64	−1.39	**−2.50**	**−2.52**	**0.07**	5.58	−4.00	0.036	U	20	10.9	6.77	0.89
	DG	17	16	0.99	0.02	4.89	−1.40	**−2.07**	**−2.17**	**0.07**	5.18	**−11.80**	0.057	U	17	10.2	6.88	0.84
	CH	25	16	0.96	0.02	4.43	−1.05	−1.37	−1.49	0.08	5.27	−5.36	**0.013**	U	25	12.1	7.69	0.88
	JP	25	15	0.90	0.05	3.80	−1.30	**−2.75**	**−2.69**	**0.07**	5.82	**−5.23**	0.025	U	25	11.7	7.28	0.86
	TU	24	13	0.92	0.04	4.04	−0.81	−0.21	−0.46	0.10	4.81	−3.85	**0.017**	U	25	10.9	7.27	0.82
	BG	25	14	0.87	0.06	3.65	−1.42	**−2.18**	**−2.28**	**0.07**	6.22	**−6.10**	0.038	U	25	11.1	7.20	0.85
	YE	18	13	0.90	0.07	3.95	***−2.19***	**−2.71**	**−2.97**	0.06^*^	7.90	**−9.29**	0.098	U	18	10.5	6.76	0.81
	NG	6	3	0.60	0.22	2.00	−1.37	−1.40	−1.49	0.30	3.00	1.02	0.204	B	3	4.1	3.64	0.81
	CAR	17	8	0.73	0.11	2.71	−0.93	−1.20	−1.30	0.11	4.44	−0.74	0.014	U	11	9.1	6.45	0.83
	CAR^a^	22	12	0.93	0.04	4.03	−0.34	−0.13	−0.23	0.11	4.02	−3.19	0.056	U	‐‐	‐‐	‐‐	‐‐
	CAR^b^	39	16	0.90	0.03	3.80	−0.85	−1.35	−1.40	0.08	5.65	−4.14	0.025	U	‐‐	‐‐	‐‐	‐‐
	ET	9	5	0.72	0.16	2.67	−1.68	−1.88	−2.04	0.14	4.55	−2.23	0.083	U	7	5.3	4.00	0.65
Group level	GH	254	68	0.91	0.01	NA	**−1.95**	**−2.67**	**−2.80**	**0.03**	17.58	**−62.45**	0.021	U	256	17.4	6.8	0.85
	WS	260	69	0.90	0.01	NA	**−1.95**	**−2.47**	**−2.68**	**0.03**	17.76	**−64.43**	0.022	U	259	17.4	5.6	0.82
	CT	299	82	0.90	0.01	NA	**−2.03**	**−2.93**	**−3.00**	**0.02**	19.87	**−87.75**	0.022	U	270	17.8	5.7	0.82
	EG	308	87	0.91	0.01	0.91	**−2.00**	**−2.69**	**−2.82**	**0.03**	19.13	**−86.15**	0.020	U	277	18.1	5.4	0.81

Notes

Sampling sites (Pop) labeled as stated in Table 1. Genetic indices of CYTB: number of sequences (*n*); number of haplotypes (*h*), haplotype diversity (*Hd*) and its standard deviation (±*SD*), and haplotype richness (*HR*). Statistical significance of the demographic statistics Class I (*Tajim* *a's* D, Fu and Li's D* and F*, Ramos‐Onsins and Rozas’ *R* *_2_*, and expansion coefficient), Class II (*F* *u's* *F*) and Class III (raggedness values) are noted in bold (*p < *0.05), bold‐italics (*p < *0.01) and bold with an asterisks (*p < *0.001). MM: mismatch distributions. Diversity indices of microsatellite markers: number of individuals genotyped (*n*); average number of alleles per locus (A); allelic richness (*A*_R_); observed heterozygosity (*H*_o_). Note small sample size of NG and ET colonies.

a CYTB sequences downloaded from GenBank.

b All CYTB sequences from Central African Republic (Museum extractions and sequences downloaded from GenBank).

The genetic diversity indices of 277 *E. gambianus* genotyped at 15 microsatellite loci (Table 2) show that the allelic richness (*A*_R_, allele numbers standardized by sample size) and observed heterozygosity (*H*_o_) of the sampled colonies are similar across the localities, having an overall *A*_R_ value of 5.4 and *H*_o_ value of 0.81. Exceptions to this are NG (*A*_R_ = 3.6) and ET (*A*_R_ = 4.0), with ET having the lowest heterozygosity of the populations (*H*_o_ = 0.65). Even though the AC colony has the lowest *A_R_* between the Ghanaian colonies, this value, along with *H*_o_, was above average across the range.

### 3.2 Demographic statistics

**Figure 3:**
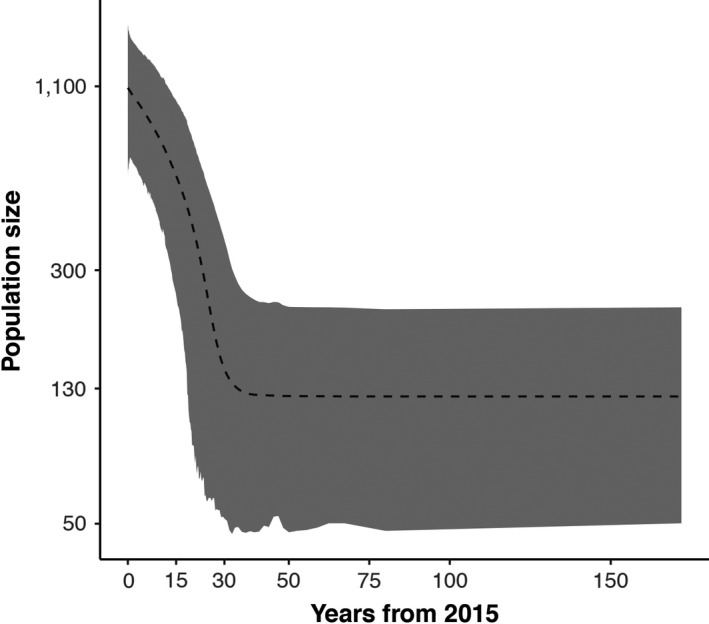
Extended Bayesian skyline plots using the concatenated mtDNA of *E. gambianus *population (including all colonies sampled). The *x* axis is in units of years before 2015, and the *y* axis (log‐scale) is equal to Neτ (product of the effective population size and the generation time in years). The dashed line is the median estimate, and the gray area displays the 95% highest posterior density

Neutrality statistics that use the mutation frequency spectrum (Class I) show a strong signature of population expansion in Ghana (Table 2), with the exception of the southern colonies of the country (AC and VG). *Tajima's D* *(D_T_)* was significantly negative in YE (*p < *0.01) and TSG (*p* < 0.05). Fu and Li's D* (D*) and F* (F*) presented significantly negative values in seven colonies within the country. Significant low positive values of Ramos‐Onsins and Rozas’ *R_2_*, and high expansion coefficients (*S/d)* correlate with previous population growth, signatures seen in the northern colonies within Ghana (all Ghanaian colonies except for AC and VG). Both AC and NG colonies have the lowest *S/d* values, which reflect a stable population size. However, no robust inferences can be made about the latter colony due to low sample size. The previous findings are corroborated by haplotype distribution statistics (Class II *Fu's F*), where large and significant negative values are shown for northern regions of the country, contrasting with the positive values of the AC and NG colonies (5.54 and 1.02, respectively). The pairwise distance statistics (Class III mismatch distributions) show that AC and NG have ragged (*rg *> 0.03) *bimodal* distributions, which reflects constant population size over a long period of time, in contrast to all other colonies that have a smooth (*rg *< 0.03) *unimodal*‐shaped distribution (reflecting rapid population growth). These results were also corroborated with coalescent extended Bayesian skyline analyses. *Epomophorus gambianus* show a rapid exponential population growth, displaying over an eightfold increase in the population in the past 30 years (Figure 3). Ghanaian populations show a constant size after a rapid increase (17‐fold) in the last 200 years, except for the colony from AC (Supporting information, Figure S5A, B) where a constant population size over time could not be rejected (NG was not analyzed due to sample size). However, the weakness of this method to small sample size and genetic structuring is reflected by the incongruence between the *y* axes of the Ghanaian versus the entire *E. gambianus *population ESBPs (Grant, Liu, Gao, & Yanagimoto, 2012). Thus, accurate historical population sizes cannot be determined. The Ghanaian population in AC is the only colony from the set that has positive values for *D_T_*, D*, and F* tests (none are significant). The combination of Class I and Class II (positive, nonsignificant values and a bimodal mismatch distribution) in this colony is consistent with a female germ line bottleneck event. In contrast to the results from the mtDNA analysis, the graphical test developed by Luikart et al. (1998) shows a normal L‐shape distribution of ncDNA alleles, which is consistent with mutation‐drift equilibrium (Supporting information Figure S6). The results from the models run in BOTTLENECK (Table 3) show no evidence of a recent bottleneck event, but instead presented significant heterozygosity deficiency (*p* value = 0.002) in the Wilcoxon test of the strict one‐step mutation model (S.M.M.), which is a signature of population growth, and there was no significance in the two‐phase model (T.P.M.). Both the S.M.M. and T.P.M. were selected due to their better analyzing microsatellite data compared to the infinite allele model (I.A.M.). The results of the M_P_Val analyses (Table 3) show that the average M ratio (0.75, *SD* = 0.19) was above the critical M value (0.688), reflecting mutation‐drift equilibrium. However, depending on the input values selected, there was evidence of a past bottleneck event (Δ*g* ≤ 2.5 when *θ* = 1–10, or Δ*g* = 3.5 when *θ* = 1), which would be consistent with the low haplotype diversity in this colony.

**Table 3:** Test of past bottleneck events in the colony of Accra

BOTTLENECK				M ratio		
Test	Models					
	T.P.M.	S.M.M.		M ratio		Mc
**Signed‐rank test**(*N*° of loci with heterozygosity excess)				0.75		0.68
Expected	10.06	10.03				
Observed	8	**4**		Values of Δ*g*		
			***θ***	*2* *.* *5*	*3* *.* *5*	
Wilcoxon test						
Heterozygosity deficiency	0.10345	**0.00158**	*1*	**0.01**	**0.47**	
Heterozygosity excess	0.90495	0.99936	*5*	**0.03**	15.43	
Heterozygosity excess or deficiency	0.20689	**0.00316**	*10*	**0.78**	37.45	

Note

Bottleneck events were tested using the software BOTTLENECK, viewing two models of mutation and showing *p* values for Wilcoxon test; and M_P_Val: assessing the M ratio (k/r), the critical *M* (Mc) and a combination of *θ* and Δ*g* input values (noted in italics) to assess the percentage in 10,000 simulations that produced equilibrium M ratio values that were significantly lower than those expected under mutation‐drift equilibrium. Values that are statistically significant (*p* < 0.05) are shown in bold.

### 3.3 Exploring population structure using F‐statistics

Genetic differentiation among populations was explored comparing pairwise *F*_ST_ and Φ_ST_ values (Table 4). With the exception of the populations from ET, AC, VG, and YE, the *F*_ST_ */*Φ_ST_ values of the studied colonies were low (<0.07) and nonsignificant, indicating free mixing of *E. gambianus* between most of the population within Ghana and as far as, and including, the Central African Republic. There was, however, a high differentiation between the Ethiopian bats and the rest of the metapopulation. Large and highly significant Φ_ST_ values ranged from 0.78 to 0.92. The AC colony had significant Φ_ST_ values with all other colonies in the analysis, except for the relatively close VG colony. Significant values (*p* < 0.01) ranged from 0.25 to 0.9, showing the greatest differentiation with the ET colonies. The VG colony also presented significant differentiation from most of the other colonies. However, compared to AC, Φ_ST_ values were overall lower (0.11–0.82) and in most cases not as highly significant (*p* < 0.05).

**Table 4:** Molecular pairwise differences between populations of *E. gambianus*

CYTB	AC	VG	TSG	BYM	TA	DG	CH	JP	TU	BG	YE	CAR	ET
AC		***0.02***	***0.02***	***0.02***	***0.02***	***0.03***	***0.01***	***0.02***	***0.03***	***0.02***	***0.02***	***0.02***	***0.06***
VG	0.00		0.00	0.01	0.01	**0.01**	0.00	0.01	***0.01***	**0.01**	0.01	0.00	***0.04***
TSG	***0.34***	***0.19***		0.00	0.00	0.00	−0.01	−0.01	0.00	0.00	−0.01	0.00	***0.03***
BYM	***0.23***	**0.11**	−0.01		0.00	0.00	0.00	−0.01	0.01	0.00	−0.01	0.00	**0.03**
TA	***0.27***	**0.13**	−0.02	−0.03		0.00	0.00	−0.01	0.00	0.00	0.00	0.00	**0.04**
DG	**0.20**	0.07	0.05	0.02	0.02		−0.01	−0.01	0.00	0.00	−0.01	0.00	0.03
CH	**0.16**	0.05	0.04	0.01	0.01	−0.02		−0.01	0.00	−0.01	0.00	0.00	**0.03**
JP	***0.25***	**0.12**	0.05	0.04	0.03	−0.02	−0.01		0.00	0.00	−0.01	0.00	0.02
TU	***0.28***	**0.14**	0.00	−0.01	−0.01	0.00	0.00	0.00		0.00	−0.01	0.00	0.01
BG	***0.35***	***0.19***	−0.02	−0.01	−0.02	0.07	0.04	**0.07**	0.01		−0.01	0.00	**0.03**
YE	***0.55***	***0.36***	0.02	**0.09**	0.06	***0.17***	***0.14***	***0.14***	**0.08**	0.04		0.00	**0.03**
CAR	***0.30***	**0.14**	−0.01	−0.02	−0.03	0.02	0.01	0.04	−0.01	−0.01	0.07		**0.04**
ET	***0.90***	***0.81***	***0.84***	***0.83***	***0.83***	***0.80***	***0.77***	***0.80***	***0.82***	***0.89***	***0.89***	***0.80***	

Notes

Pairwise mtDNA (CYTB) Φ_ST_ values are shown below the diagonal and pairwise ncDNA (microsatellite) *F*_ST_ values are shown above the diagonal. *p* values are corrected for multiple testing, where *p < *0.05 is denoted in bold and *p* < 0.01 is presented in bold and italicized values. Only SI samples were used for ET.

*F*_ST_ values were much lower than Φ_ST_ values; nevertheless, pairwise *F*_ST_ values indicated the same structural pattern as did the Φ_ST_ estimates, except for the colony in Yendi. There is significant structure between the ET colonies and the others, as well as AC and all the other colonies (Table 4). Additionally, VG had a low but significant (*p* < 0.05) differentiation with several other Ghanaian populations.

A hierarchical AMOVA was used to test the results for both Φ_ST_ and *F*_ST_ at the population and group levels (Table 5), further exploring the results from Table 4. When Ghanaian populations were grouped separately from the rest of the bat colonies, the percentage of variation between populations decreased from 27.2% to 9.4% (Table 5, *analyses 1 and 2*). When both AC and VG were removed from the Ghanaian group (rGHANA, Table 5, *3*), it resulted in the lowest Φ_ST_ value (0.02) obtained for any combination tested, indicating panmixia among the remaining colonies. The same pattern was detected among rGHANA populations using microsatellite markers *F*_ST_ values, showing complete panmixia with almost 100% of the variability explained within bat colonies (Table 5, *3*). The high fixation values (Φ_ST_ *=* 0.8) among ET, CAR, and NG (Table 5, *4*) were mostly explained by variation among the grouped colonies (80%). Supporting this result, the analysis of grouped CAR, NG, and Ghanaian colonies against ET (Table 5, *7*) revealed that the highest variation was explained among groups (Φ_CT_ = 0.79) with moderate Φ_SC_ values (structure among populations within groups).

**Table 5:** Hierarchical AMOVA analysis and population structure using: mtDNA CYTB (Φ*‐*statistics) and ncDNA microsatellites (*F‐*statistics)

Structure tested	% Variance	Φ*‐*statistics		*p*	% Variance	*F* *‐*statistics		*p*
1. One group (all populations)								
*Among populations*	27.2				1.5			
*Within populations*	72.8	Φ_ST_	0.27	**0.00**	98.5	*F*_ST_	0.01	**0.00**
2. One group (Ghanaian populations)								
*Among populations*	9.4				0.8			
*Within populations*	90.6	Φ_ST_	0.09	**0.00**	99.2	*F*_ST_	0.01	**0.00**
3. One group (Ghana excluding AC and VG)								
*Among populations*	2.5	Φ_ST_	0.02	**0.01**	0.3			**0.01**
*Within populations*	97.5				99.7	*F*_ST_	0.00	
4. One group (NG + CAR + ET)								
*Among populations*	80.1				11.6			
*Within populations*	19.9	Φ_ST_	0.80	**0.00**	88.4	*F*_ST_	0.12	**0.00**
5. Two groups (Ghana) versus (NG + CAR + ET)								
*Among groups*	*14* *.* *5*	Φ_CT_	0.14	0.13	0.9	*F*_CT_	0.01	0.13
*Among populations*	*21* *.* *0*	Φ_SC_	0.25	**0.00**	1.3	*F*_SC_	0.01	**0.00**
*Within populations*	*64* *.* *6*	Φ_ST_	0.35	**0.00**	97.8	*F*_ST_	0.02	**0.00**
6. Two groups (Ghana + NG) versus (CAR + ET)								
*Among groups*	22.6	Φ_CT_	0.23	0.25	0.9	*F*_CT_	0.01	0.25
*Among populations*	18.1	Φ_SC_	0.23	**0.00**	1.33	*F*_SC_	0.01	**0.00**
*Within populations*	59.3	Φ_ST_	0.41	**0.00**	97.77	*F*_ST_	0.02	**0.00**
7. Two groups (Ghana + NG + CAR) versus (ET)								
*Among groups*	79.1	Φ_CT_	0.79	0.14	5.38	*F*_CT_	0.05	0.14
*Among populations*	1.9	Φ_SC_	0.09	**0.00**	1.1	*F*_SC_	0.01	**0.00**
*Within populations*	19.0	Φ_ST_	0.81	**0.00**	93.52	*F*_ST_	0.06	**0.00**
8. Two groups (AC + VG) versus (rGHANA)								
*Among groups*	18.1	Φ_CT_	0.18	**0.02**	0.96	*F*_CT_	0.01	**0.02**
*Among populations*	2.0	Φ_SC_	0.02	**0.04**	0.42	*F*_SC_	0.00	**0.00**
*Within populations*	79.9	Φ*_ST_*	0.20	**0.00**	98.62	*F*_ST_	0.01	**0.00**
9. Three groups (Ghana) versus (NG + CAR) versus (ET)								
*Among groups*	52.6	Φ_CT_	0.53	0.06	2.26	*F*_CT_	0.02	0.06
*Among populations*	4.4	Φ_SC_	0.09	**0.00**	1.07	*F*_SC_	0.01	**0.00**
*Within populations*	43.0	Φ_ST_	0.57	**0.00**	96.67	*F*_ST_	0.03	**0.00**
10. Four groups (AC + VG) versus (rGHANA) versus (NG + CAR) versus (ET)								
*Among groups*	41.0	Φ_CT_	0.41	**0.00**	1.45	*F*_CT_	0.02	**0.02**
*Among populations*	1.4	Φ_SC_	0.02	0.07	0.8	*F*_SC_	0.01	**0.00**
*Within populations*	57.6	Φ_ST_	0.42	**0.00**	97.75	*F*_ST_	0.02	**0.00**

Notes

*p *Values (*p*) below 0.05 are noted in bold. rGHANA: Ghanaian colonies excluding AC and VG. Note the small sample size of NG and populations of ET.

As previously described, our analysis of microsatellite markers supports the mtDNA findings, although the differentiation between the groupings and the resulting *F*_ST_ values are not so decisive. Particularly, no great difference is detected when grouping Ghana by itself against NG, CAR, and ET combined, or when grouping Ghana and NG combined against CAR and ET combined (Table 5, *analyses 5 and 6*). This might be due to the very small genotyping sample size for the Nigerian colony. These results were explored with additional more sensitive statistics, which were consistent with the *F*_ST_ findings (Supporting information Table S7). In addition, to evaluate the bias produced by both the small sample size of NG and ET and the colonies geographical distance of separation, analyses were run excluding the bats from NG and DI colonies. Results display equivalent structuring patterns (Supporting information Table S8).

To determine that the computed low Φ_ST_ or *F*_ST_ values were not a result of insufficient sample size, power calculations were conducted with *F*_ST_ values ranging from 0.001 to 0.01. This showed that the microsatellite dataset provided 100% power to detect population structuring when *F*_ST_ = 0.0035%, and 82% if the true *F*_ST_ was 0.002. Mitochondrial markers provided 100% power to detect population structure when the true Φ_ST_ value was 0.005%, and 81% when Φ_ST_ = 0.003. In the AMOVA analyses, the minimum Φ*_ST_* and *F*_ST_ values were 0.02 and 0.003, respectively, which should be detected with <80% power. The estimated occurrence of a type 1 error (α) in the dataset ranged from 0.05 to 0.07, which suggests acceptable performance.

### 3.4 Isolation by distance

When analyzing the correlation between the logarithmic geographic distance and the genetic distance, either as linearized Φ_ST_ for the CYTB (Supporting information Figure S9A–E) or linearized *F*_ST_ for the microsatellite markers (Supporting information Figure S9F–J), a positive significant correlation was detected when all the populations of *E. gambianus* were included (mtDNA: *R* ^2^ = 0.4, *p* value <0.05; ncDNA: *R* ^2^ = 0.2, *p* value <0.05). But after excluding the Ethiopian colony from the analyses, no correlations were detected. The same non‐significant result was found after removing CAR and/or NG. Within Ghanaian colonies, there was a significant positive correlation with both mtDNA: *R* ^2^ = 0.2, *p* value <0.001 and ncDNA: *R* ^2^ = 0.3, *p* value <0.01, but after excluding the colonies AC and VG from the analysis, the significance was lost. However, when only excluding AC from the Ghanaian colonies, significant values were still detected for both molecular markers.

### 3.5 Exploring population structure using Bayesian clustering methods—ncDNA

**Figure 4:**
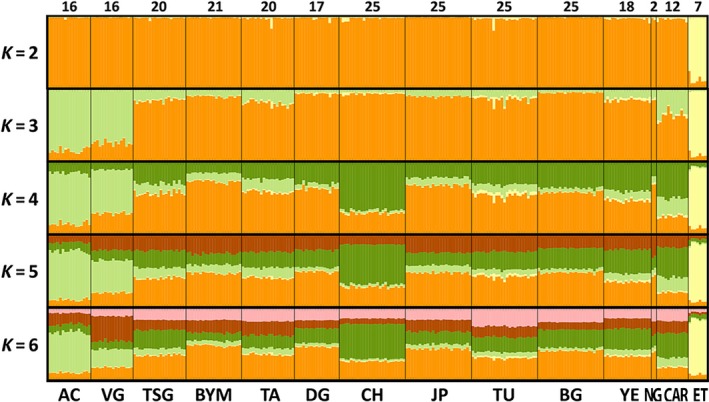
STRUCTURE analysis of 249 specimens using an Admixture model, correlated allele frequencies, and LOCPRIOR. Populations are separated with thick black lines and labeled at the bottom by the colony IDs stated previously in Table 1. NG inferences are not robust due to sample size

All models and datasets that were tested with STRUCTURE clustered the populations from AC, VG, and ET (Figure 4; Supporting information Figure S10). The model using the averaged sample numbers (total of 249 bats) showed, in 100% of the replicated alignments, a clear differentiation between ET and the rest of the colonies (K = 2). By incrementing one more cluster, AC and VG emerged as a separate population (Figure 4); no other populations were robustly segregated with increasing values of K. Modeling the dataset with the total number of samples (Supporting information Figure S10, 277 bats), AC and VG segregate at *K* = 2 in 90% of the replicated alignments with ET emerging in the subsequent division; the remaining 10% resulted in ET segregating first, following the previous model pattern. The highest number of clusters, selected by Delta K, that best explains the data for both models was *K* = 4 (Supporting information Figure S11).

### 3.6 Exploring population structure using intraspecific phylogeny—mtDNA

**Figure 5:**
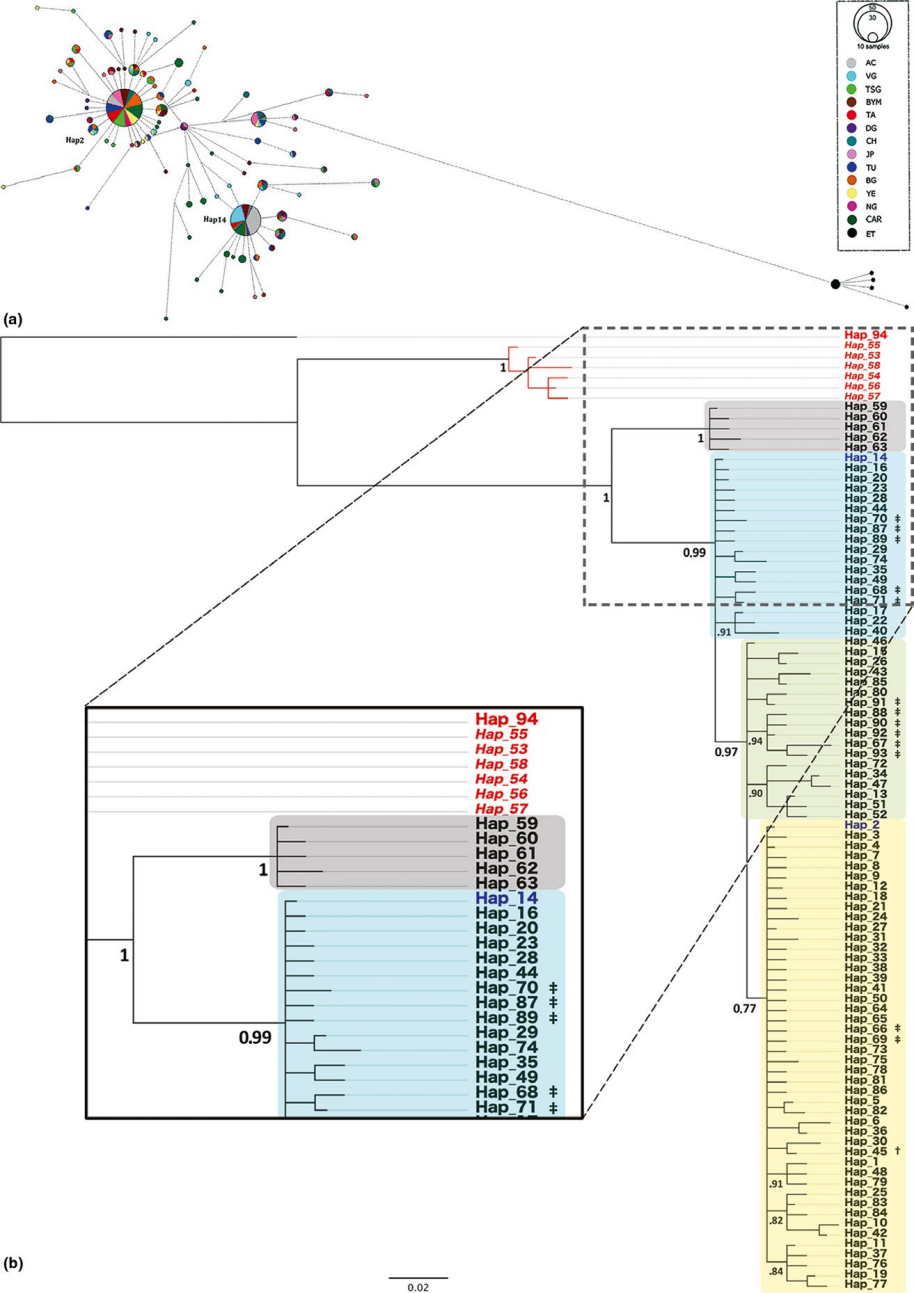
*Epomophorus gambianus *systematics. (a) Median‐joining haplotype network. The circles represent unique haplotypes of CYTB sequences*.* Circle size is proportional to the frequency of specimens sharing that haplotype, and the color reflects the population of origin. The lines between two haplotypes show base substitutions, and its length is proportional to the number of point mutations. There is a clear spatial clustering between the Ethiopian colony (in black) and the rest of the African populations. (b) Bayesian phylogeny of *E. gambianus *CYTB haplotype alignment. Out‐group species are labeled in red: *Rousettus aegyptiacus* (Hap 94) and *Epomops franqueti* (Hap 53–58). There are two distinct clades in the *E. gambianus* phylogeny, one monophyletic group generated by the Ethiopian population (black box) and the rest (subdivisions highlighted with colored boxes). Haplotypes 2 and 14 are typed in blue. Private haplotypes to Nigeria (†) and to Central African Republic (‡) are denoted. Posterior probabilities are shown above the main nodes, and estimated divergence time (Mya) between clades is shown within brackets

The haplotype network of the CYTB fragment of *E. gambianus* (Figure 5a) is characterized by two common “central” haplotypes (Hap2 and Hap14), a few less‐common ones and a large number of rare “external” haplotypes. Population expansion can be inferred by the distribution of the *external* haplotypes diverging in a star‐like topology from the *central* ones. Colonies grouped at the country‐level (Supporting information Figure S12A) better demonstrate the geographical extent of gene flow of CYTB haplotypes. In addition, the D‐loop haplotype network (Supporting information Figure S12B) shows a consistent geographical pattern with CYTB at the colony level as well as the individual level (by the same specific bats). The important features of the CYTB haplotype network (Figure 5a) are as follows:

1. Far in the right lower corner (separated by 16 nucleotide substitutions), there are five “private” haplotypes, found only within the Ethiopian samples. Even though there is a likely significant bias caused by these heterochronous samples (42 years apart to Ghanaian sequences), this geographical divergence was not present in other museum‐acquired specimens (18 years apart, Table 1).
2. The two *central* haplotypes are formed by individuals from all the colonies sampled with the exception of ET, indicating relatively free mixing throughout the geographical range of *E. gambianus* apart from Ethiopia.
3. Accra bats are only represented in the two common “central” haplotypes (Hap2 =24%, Hap14 =76%). Interestingly, all of the colonies except for VG contain the two central haplotypes and only Hap14 is represented in both AC and VG.

The intra‐ and interspecific relationships of *E. gambianus* populations and out‐group species (*Epomops franqueti* and *Rousettus aegyptiacus*) were analyzed using an alignment of 94 CYTB haplotypes (with 139 variable sites) found in the 328 sequences aligned and of 200 haplotypes (267 variable sites) found in the concatenated fragment (from 301 sequences). These are shown using alternative substitution models, both for the CYTB (Figure 5b; Supporting information Figure S3A) and concatenated alignment (Supporting information Figure S13A, B) Bayesian phylogenetic trees, as well as for the CYTB maximum‐likelihood trees (Supporting information Figure S3B, D). Consistent with previous phylogeographic classifications (Almeida, Giannini, DeSalle, & Simmons, 2011), both *Rousettus aegyptiacus* (used as the root of the tree) and *Epomops franqueti* are ancestral to *E. gambianus.* The intraspecific patterns shown in the phylogenetic tree corroborate the phylogeographic structure of the haplotype network: The Ethiopian bats (clade highlighted in black) are divergent from all other populations of *E. gambianus* sampled. A further subdivision of the non‐Ethiopian *E. gambianus* is shown in the three trees, separating haplotypes 14 and 2. However, there is no robust support for these branches. The estimated divergence time of the ET colonies and the rest of the *E. gambianus* population is approximately 1.6–1.8 Mya (Figure 5b).

## 4 DISCUSSION

While several species of bat have been shown previously to be panmictic (Peel et al., 2013; Russell et al., 2005; Sinclair, Webb, Marchant, & Tidemann, 1996; Webb & Tidemann, 1996), genetic structuring within a species can vary due to various dispersal or migratory behaviors (Fleming, Murray, & Carstens, 2010; Moussy et al., 2013). Several studies exemplify male, but not female, panmixia in bats (Kerth, Mayer, & Petit, 2002; Rossiter et al., 2002; Rydell, 1989); however, female‐biased bat dispersal has been reported (Nagy et al., 2007). By comparing the fixation indices between uniparental and biparental markers, it is possible to estimate the extent of sex‐biased dispersal (Prugnolle & de Meeus, 2002). In the current study, we show that across most of the range of *E. gambianus,* there is congruent phylogeographic structuring between both maternally and biparentally inherited markers, which suggests long‐range dispersal of both sexes. *Epomophorus gambianus* connectivity and free mixing were demonstrated across the colonies sampled throughout most of its distribution. Besides the peripheral Ethiopia, Accra and, in some degree, Ve‐Golokwati colonies, there was no evidence of an IBD pattern of dispersal between the colonies. There is, however, a signal of genetic differentiation in the female line of descent, which is not explained by the expected conflict between hemizygous mtDNA and multiallelic ncDNA markers (Birky, Maruyama, & Fuerst, 1983), indicating that male dispersal occurs more frequently and/or over longer distances than female dispersal. It was also possible to infer past population expansions and bottleneck events.

The Ethiopian colony was consistently identified as a divergent population. These bats were surrounded by the Ethiopian highlands (over 3,000 m asl) and lakes with over 129 km^2^ of surface (Ethiopian Mapping Authority (EMA) 1988), which likely disrupted gene flow between this colony and the rest of the *E. gambianus* population. Even though in some studies, mountains can be considered a weak obstacle for flying taxa (Demont, Blanckenhorn, Hosken, & Garner, 2008; Moussy et al., 2015; Petit & Mayer, 1999; Xu et al., 2010), this distance combined with numerous water bodies seems an effective geographical barrier for this lowland bat species. In addition, the phylogeny of *E. gambianus* revealed that the Ethiopian bats diverged over ~1.6 Mya to the rest; however, the age of our samples is likely overestimating this divergence. It has been hypothesized that an Asian ancestor of the myonycterine‐epomophorine clade colonized Africa through the forested corridors that linked Asia and Africa (prior to the rise of the mountains), with consequent evolutionary radiation (Juste et al., 1999). Therefore, the colonization of *E. gambianus* could have followed an east–west axis. However, other dispersal routes or vicariance events, that may explain this phylogenetic pattern, cannot be ruled out. Furthermore, the series of ice ages that occurred during the Pleistocene and upper Pliocene (~3.5 Mya–12,000 years ago) were associated with processes of forest contraction and habitat fragmentation that shaped the vegetation and forest systems present in ancient Africa (Hamilton & Taylor, 1991; Hewitt, 2000). During this era, the fragmented patches of refugia drove widespread speciation and divergence during three progressive climate shifts that increased arid conditions (deMenocal, 1995). The second one (1.8–1.6 Mya), associated with speciation by isolation of bat species within the tribe Myonycterini (Nesi et al., 2013), could have influenced populations of *E. gambianus*, resulting in the strong genetic differentiation of the possibly ancestral Ethiopian lineage.

The southern colonies within Ghana (Accra and Ve‐Golokwati) had moderate and predominantly female‐biased genetic structuring compared to the rest of the populations. While the flying capabilities of bats usually secure free dispersal, water bodies have disrupted gene flow in both insectivorous (Castella et al., 2000; García‐Mudarra, Ibáñez, & Juste, 2009) and frugivorous (Peel et al., 2013) bat species. The Volta River and Lake Volta, one of the biggest water bodies in West Africa (8,500 km^2^ of surface), are situated in the Volta basin, which lies to the north and east of Accra and Ve‐Golokwati. Even though *E. gambianus* is likely to be able to fly across the Volta river, genetic divergence due to rivers (smaller in width than the bats’ average foraging flight distance) has been reported for other bat species, including the insectivorous *Eptesicus serotinus* (Moussy et al., 2015) and the frugivorous *Scotonycteris bergmansi* (Hassanin et al., 2015).

The demographic history of *E. gambianus* shows both spatial and demographic expansion in most of its colonies, which is consistent with a sudden population growth after reduced population sizes (Grant, 1998) and previously recognized expansion signatures of bats after colonizations from vicariance periods (Juste et al., 1999; Petit & Mayer, 1999). An exception is the colony of Accra, where the strong genetic signature reflects a past female germ line bottleneck. Here, *E. gambianus* coroosts with a conspicuous and large population of *E. helvum.* Bottleneck events can rapidly reduce variability in species, like *E. gambianus*, that have long generation lengths and low reproductive outputs (O'Brien & Hayden, 2004). However, contrary to the mitochondrial information, nuclear DNA did not support a bottleneck event (Luikart et al., 1998). Using the BOTTLENECK software, the “standardized differences test” and the I.A.M. model were excluded due to their low statistical power and unsuitability for microsatellite data. The most powerful test to analyze less than 20 loci is the “Wilcoxon signed‐rank test,” which detected a significant heterozygosity deficiency in this dataset, suggesting population expansion instead of decline (Cornuet & Luikart, 1996). However, the detected high frequency of a few alleles could suggest an ancient bottleneck and the heterozygosity deficiency could reflect inbreeding (Wright, 1921), nonrandom sampling of family members (Luikart & Cornuet, 1999) or false expansion signals often detected in IBD structures (Leblois, Estoup, & Streiff, 2006). The M ratio was also analyzed, as it performs better at detecting past bottleneck events than the BOTTLENECK algorithm (Peery et al., 2012; Piry et al., 1999). However, no ancient bottleneck signature was identified with the parameters selected and the results obtained with a range of input values were inconsistent, likely due to the known sensibility of this model to the parameter assumptions and IBD (Leblois et al., 2006). This conflict between markers could be due to the fourfold difference in effective population size from the haploid mitochondria, generating bottleneck signatures in the mtDNA from smaller reductions of the population size and/or an ancient brief bottleneck event, which would not be evidenced otherwise by the ncDNA (Birky, 1991; Wilson et al., 1985). Also, as the nuclear microsatellite markers have higher mutation rates, a bottleneck signal could have already been erased from them (Cornuet & Luikart, 1996; Rogers, 1995). Furthermore, selective sweeps (Maruyama & Birky, 1991), founder events (Ashley & Wills, 1987), stochastic lineage extinctions (Avise, Neigel, & Arnold, 1984), or lack of power for detecting recent bottlenecks (Peery et al., 2012) cannot be ruled out.

Likely the habitat disturbance due to the rapidly expanding urbanization of Accra and/or the still unknown effects that social dynamics with conspecifics have to roost integrity (Kunz & Fenton, 2003) could have driven a decline (via migration, hunting, etc.) and genetic bottleneck in the Accra population. Furthermore, there is a clear association between the Accra and Ve‐Golokwati colonies, perhaps reflecting selective connectedness due to the isolation with the other colonies due to an extensive forest cover loss (Supporting information Figure S14; Hansen et al., 2013). Nevertheless, a past founder event from Ve‐Golokwati or the continued connectedness between the colonies cannot be disregarded. Fossil evidence dating 21,000–12,000 years ago shows that vegetation zones in lowland Ghana (below the Volta basin) were depressed at least for several hundred meters of altitude and the area presented major forest reduction at glacial maximum (Hamilton & Taylor, 1991). This forest loss and fragmentation possibly shaped the genetic signatures of both the Accra and Ve‐Golokwati colonies. The extremely limited haplotype richness in Accra despite a strong central continent connectivity (Supporting information Figure S15), however, suggests a more recent bottleneck or founder effect. As the time of divergence was assessed with general clock rates of mammalian mtDNA used at the speciation level and the substitution rates at the species level differ greatly to that of intraspecific divergence (Ho, Saarma, Barnett, Haile, & Shapiro, 2008), it was not possible to accurately evaluate the time of recent demographic events, particularly of nearby colonies within Ghana.

## 5 CONCLUSIONS

The results presented in this study confirm connectivity and free gene flow of *E. gambianus* across much of its range. Panmixia was demonstrated throughout the Central African Republic to the northern and central regions of Ghana. Between these colonies, there was no evidence of population divergence due to geographical isolation or preferential breeding, although males seem to disperse longer distances or more frequently. In contrast, the Ethiopian colony of *E. gambianus* is genetically divergent from the rest of its population. Complementary studies using homochromous samples would greatly benefit the evaluation of the extent of this divergence. The Ghanaian lowland colonies sampled also show genetic differentiation from the rest of the other sampled colonies, with a strong genetic signature of a past bottleneck in the female line of descent in the Greater Accra colony. Both the geographical landscape and the species ecology suggest that the geographical barriers (mountains and water bodies) and other environmental developments (e.g., urbanization of megacities) evaluated in this research are likely drivers of the regional genetic divergences.

## CONFLICT OF INTEREST

None declared.

## AUTHOR CONTRIBUTIONS

Silke Riesle‐Sbarbaro designed and performed the research, analyzed the data, and wrote the first draft of the manuscript. Kofi Amponsah‐Mensah contributed in sampling methodology and acquired bat samples. Stefan de Vries supervised the development of nuclear microsatellite primers and contributed reagents (Illumina). Violaine Nicolas contributed in acquiring bat samples and extracted DNA samples. Aude Lalis contributed in acquiring bat samples and extracted DNA samples. Richard Suu‐Ire contributed in acquiring bat samples. Andrew A. Cunningham designed and supervised the research and reviewed and edited the manuscript. James L. N. Wood designed and supervised the research and reviewed and edited the manuscript. David Sargan designed and supervised the research, including the laboratory techniques and the experimental designs; reviewed and edited the manuscript; and contributed reagents. All authors improved the manuscript.

## DATA ACCESSIBILITY

The mitochondrial DNA alignments used in this study, the nuclear microsatellite RAW data (.fsa) and the outputs of the STRUCTURE analysis performed using 277 bats, are included in Dryad Digital Repository: https://doi.org/10.5061/dryad.m8m6142. Mitochondrial DNA alignments will be also included in GenBank. Primers for both mitochondrial DNA and nuclear microsatellite markers are included in the Supplemental Information.
