# Supplementary material for: The Gambian epauletted fruit bat shows increased genetic divergence in the Ethiopian highlands and in an area of rapid urbanization
Source: Ecol Evol. 2018 Dec 11;8(24):12803–20. doi: 10.1002/ece3.4709 (PMC6308866; doi:10.1002/ece3.4709)
Supplement: Supplementary file 1 [file ECE3-8-12803-s001.docx]

**Supplemental Information for:**

**The Gambian epauletted fruit bat shows increased genetic divergence in the Ethiopian highlands and in an area of rapid urbanisation.**

Silke Riesle-Sbárbaro, Kofi Amponsah-Mensah^,^ Stefan de Vries, Violain Nicolas, Aude Lalis, Richard Suu-Ire, Andrew A. Cunningham, James L. N. Wood and David Sargan^.^

**Table of Contents:**

| **Table S1** | Page 1 |
| --- | --- |
| **Table S2** | Page 2 |
| **Fig. S3 A** | Page 3 |
| **Fig. S3 B** | Page 4 |
| **Fig. S3 C** | Page 5 |
| **Fig. S3 D** | Page 6 |
| **Table S4** | Page 7 |
| **Fig. S5** | Page 8 |
| **Fig. S6** | Page 9 |
| **Table S7** | Page 9 |
| **Table S8** | Page 10 |
| **Fig. S9** | Page 11 |
| **Fig. S9 Continuation** | Page 12 |
| **Fig. S10** | Page 13 |
| **Fig. S11** | Page 14 |
| **Fig. S12** | Page 15 |
| **Fig. S13 A** | Page 16 |
| **Fig. S13 B** | Page 17 |
| **Fig. S14** | Page 18 |
| **Fig. S15** | Page 19 |

**Table S1. Primer pairs used to amplify mitochondrial DNA markers**.

| **Primer ID** | **Primer sequence 5’ to 3’** |
| --- | --- |
| D loop | F: TTGTAAACCAGAAAAGGGGAAT R: ATACCAGAGGCATGACACCA |
| CYTB | F: GACTTATGGCATGAAAAACCAC R: GATTCCGGTGGGATTATTTG |

**Table S2. Characterization of 20 nuclear microsatellite markers used in this study.**

| Mix ID | Locus ID | Primer sequence (5'-3')*^a^* | *n* | Allele size range (bp) | *H_o_* | *H_e_* | Repeat motif |
| --- | --- | --- | --- | --- | --- | --- | --- |
| mA | **L3** | F: T7-TCTCCAGGAATCTGTCCTCAC R: TTGCTGGATTTGATCCACTG | 27 | 244-302 | 0.89 | 0.89 | (CA)*_n_* |
|  | **L8** | F: T7-GCACCTCCCTGGTAGTCTCA R: CCCTGACTGCTCTTTTCAGG | 16 | 210-251 | 0.87 | 0.88 | (CA)*_n_* |
|  | **L17** | F: M13-TGGCTCCTTTTATGTGCTAGG R: GATGCTGAGCTTTTGAATCG | 27 | 230-297 | 0.87 | 0.91 | (CA)*_n_* |
|  | **L23** | F: M13-AGCCAAGATATGGAGACATCCT R: TCTGTTTGATACTCCCCACTCA | 16 | 180-232 | 0.85 | 0.88 | (AC)*_n_* |
|  | **L32** | F: T3-TCAAACCTATCTCTTCTGGAATG R: GTGGCTCTCCACCCTAGC | 11 | 179-223 | 0.82 | 0.83 | (GATA)*_n_* |
| mB | **L2** | F: T7-GTTTTCCAAATGCTGGCTTC R: AGGTTTGTGGTGGAGTCAGG | 19 | 228-266 | 0.87 | 0.89 | (CA)*_n_* |
|  | **L25*^b^*** | F: M13-TGAATTAACAACCCTCCTCTCTG R: CCATGCGTCTAGATTCAGCA | 17 | 227-269 | 0.43 | 0.51 | (AC)*_n_* |
|  | **L31** | F: T3-CCGGGTTTCCTTAGATTTCTG R: AACCCTTGGTCAGTCACCTG | 11 | 215-247 | 0.62 | 0.68 | (CA)*_n_* |
|  | **L35** | F: T3-GGCATTGTGTGCCTTGTG R: TCAGGAGCTGATGATTCCAC | 14 | 300-342 | 0.89 | 0.87 | (AC)*_n_* |
| mC | **L5** | F: T7-TACCTTCTGGAGGTGGGATG R: CATCGTACCATGTTGCTTGC | 12 | 219-245 | 0.82 | 0.81 | (CA)*_n_* |
|  | **L7** | F: T7-CCTCTTCCCATGAAAATACTTAACA  R: GGCAGGGTAGTCTGCATGTAA | 20 | 249-283 | 0.89 | 0.92 | (AC)*_n_* |
|  | **L26*^b^*** | F: M13-CCTTCGAGATTATCCTGGCTAT R: GGAGGCATATGTCAAGTCTCG | 14 | 228-270 | 0.69 | 0.87 | (AC)*_n_* |
|  | **L34** | F: T3-TTTGTTTGTGTTTCTTGTCTGTTATG R: GGACTGGGCCCAATAATAAAG | 16 | 194-234 | 0.83 | 0.85 | (AC)*_n_* |
| mD | **L15** | F: SP6-GTGCCAGGCTTTCTGGATT R: TCAACCTTCACTTTTCCTCCA | 16 | 186-222 | 0.81 | 0.89 | (AC)*_n_* |
|  | **L24** | F: M13-TTGCCAGTGAACTAGTGACCA R: GATAGCCTGGAGCAAAGTGG | 21 | 183-231 | 0.85 | 0.89 | (AC)*_n_* |
|  | **L30** | F: T3-CATTTGTGGGCAGTGGTATG R: TGAGCAGGAAGAGGAAGCAG | 13 | 203-235 | 0.85 | 0.86 | (CA)*_n_* |
|  | **L36*^b^*** | F: T7-GTGCCTCAATCCTACAAGGTG  R: TGTGTTTGTCTCATATGTGTGTGTC | 14 | 218-246 | 0.73 | 0.88 | (AC)*_n_* |
| mE | **L6** | F: T7-CAAAGAATTCAGGGATTACAAAAG R: GGGCATAAATACCATCAAAGTG | 15 | 218-248 | 0.84 | 0.91 | (AC)*_n_* |
|  | **L12** | F: SP6-TGCTCTGTTTTAGCCTTCTGC R: CACCTGCTTTAAAGATCATTTTCTC | 25 | 175-233 | 0.89 | 0.89 | (AC)*_n_* |
|  | **L29** | F: T3-AGCCAACATATGGAAACAATCT R: CACTTAGCATAATATCTTGTAGGTTCA | 20 | 200-278 | 0.85 | 0.86 | (CA)*_n_* |

*n:* total number of alleles per locus; *H_o_*: Observed heterozygosity; *H_e_*: Expected heterozygosity; *a*: Fluorescent dyes hybridised to the universal primers: T7 (TAATACGACTCACTATAGGG), SP6 (ATTTAGGTGACACTATAGAA), M13 (CACGACGTTGTAAAACGAC), T3 (ATTAACCCTCACTAAAGGGA); *b*: Loci with significant deviation from HWE in two populations (BYM-CAR), p<0.05 adjusted with FDR correction.

**Fig. S3A Bayesian phylogeny of *Epomophorus gambianus* CYTB haplotype alignment (532 bp), using a GTR+G model and sampled for 10^9^ generations. Hap 2 and 14 are typed in blue. The ET clade is noted in bold and with a black line. Outgroups are labelled in red for *E. franqueti* (Hap 53 to 58) and with an asterisk for *R. aegyptiacus*.**

*****

**
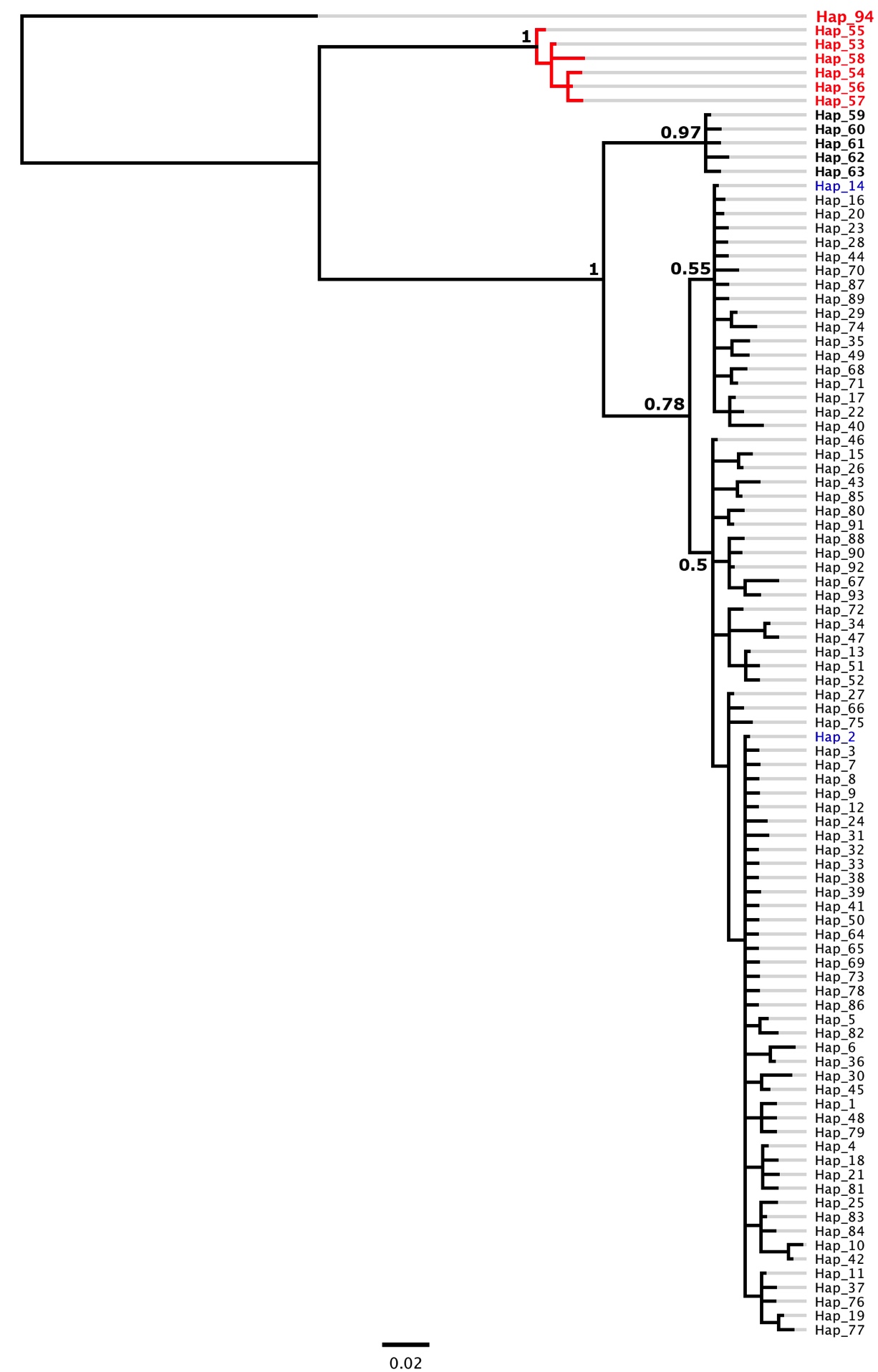
**

**Fig. S3B Maximum likelihood phylogeny of *Epomophorus gambianus* CYTB haplotype alignment (532 bp), using the HKY+G model and 1000 bootstrapped iterations. The ET clade is noted in bold and with a black line. Hap 2 and 14 are typed in blue. Outgroups are labelled in red: *E. franqueti* (Hap 53 to 58) and with an asterisk: *R. aegyptiacus*.**

*****

**
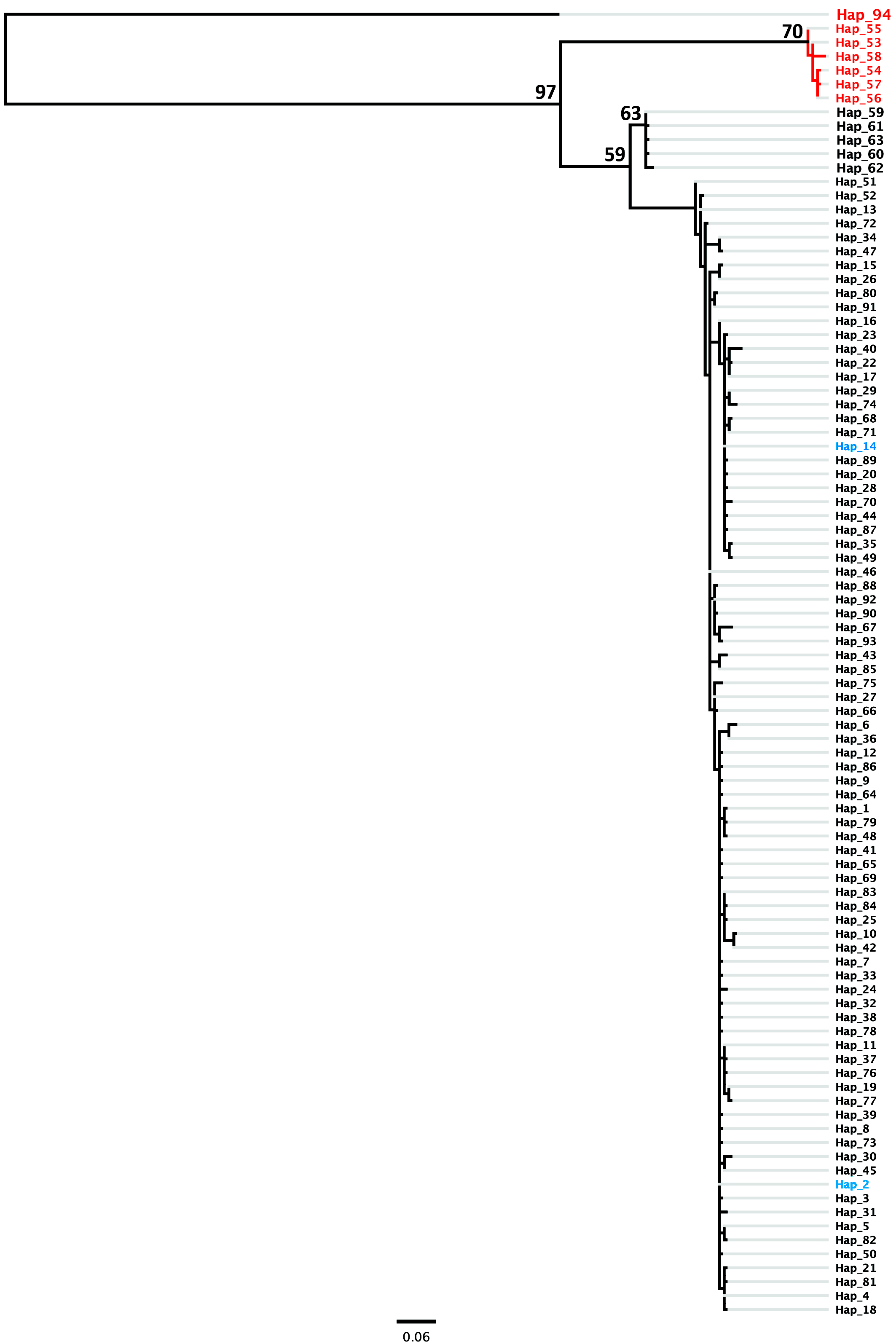
**

**Fig. S3C Maximum likelihood phylogeny of *Epomophorus gambianus* CYTB haplotype alignment (532 bp), using the K80 model and 1000 bootstrapped iterations. Hap 2 and 14 are typed in blue. The ET clade is noted in bold and with a black line. Outgroups are labelled in red: *E. franqueti* (Hap 53 to 58) and with an asterisk: *R. aegyptiacus*.**

*****

**
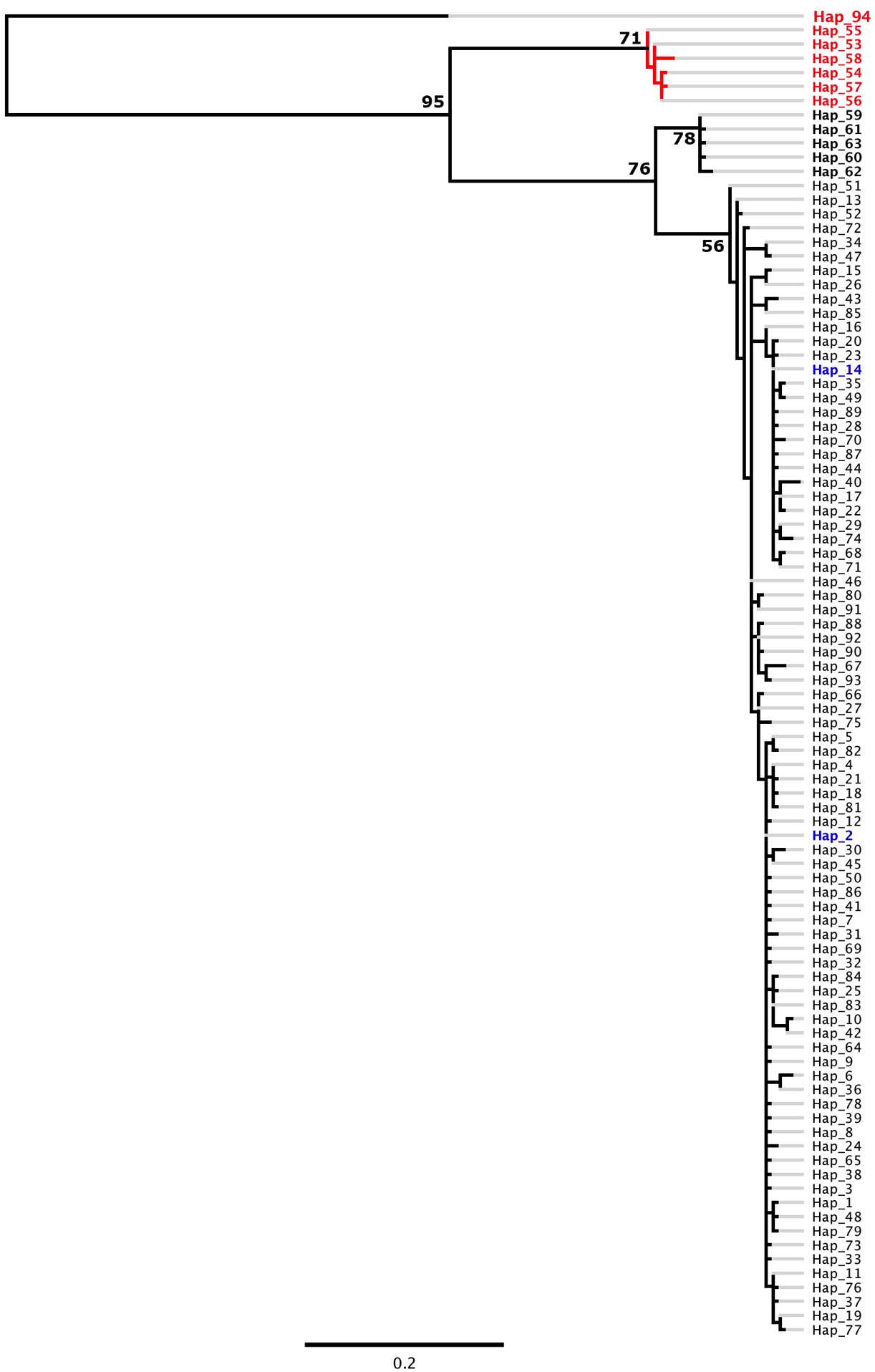
**

**Fig. S3D Maximum likelihood phylogeny of *Epomophorus gambianus* CYTB haplotype alignment (532 bp), using the TN93 model and 1000 bootstrapped iterations. Hap 2 and 14 are typed in blue. The ET clade is noted in bold and with a black line. Outgroups are labelled in red: *E. franqueti* (Hap 53 to 58) and with an asterisk: *R. aegyptiacus*.**

*****

**
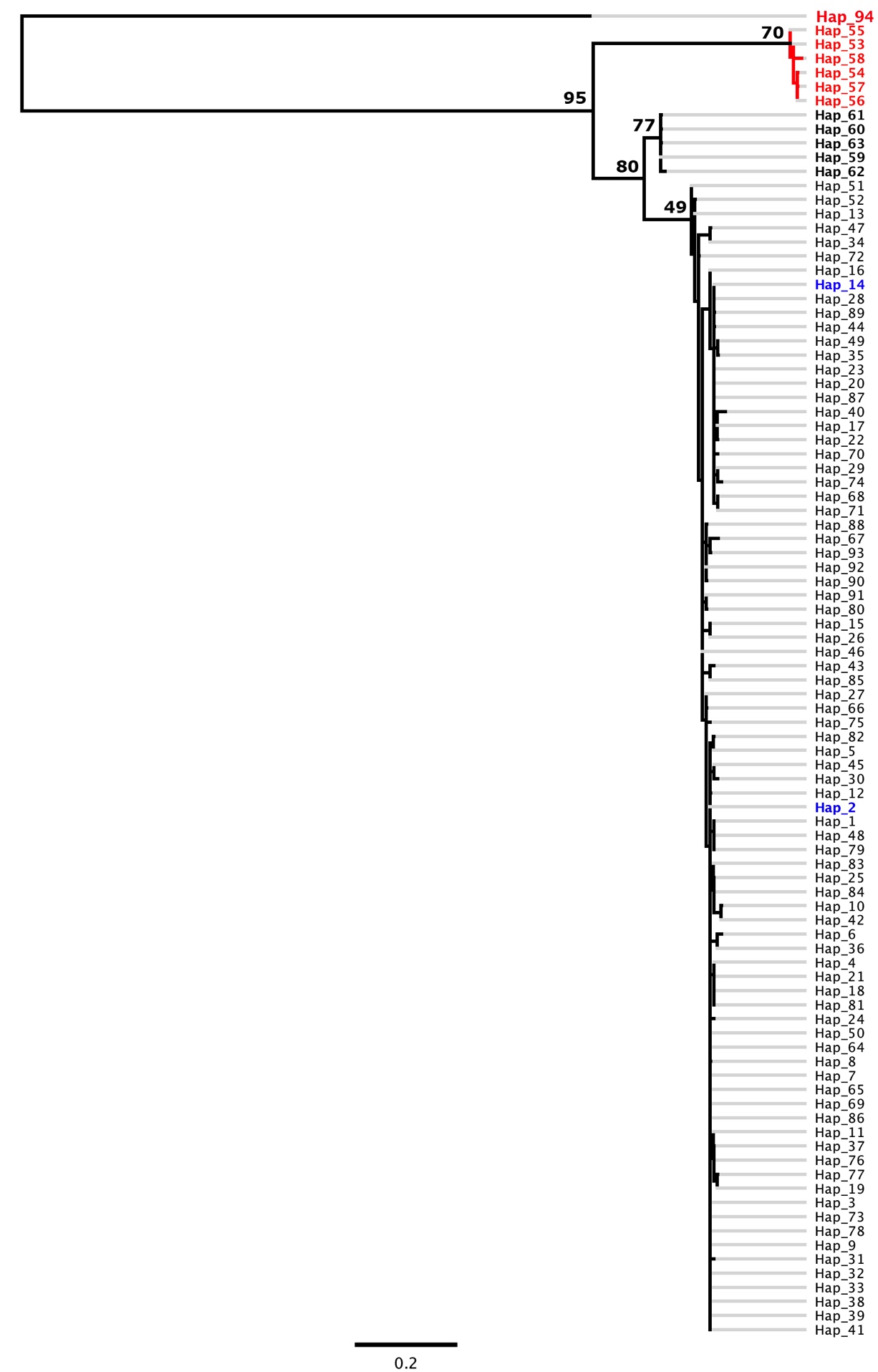
**

Table S4. Modified weightings for hypervariable nucleotide sites used in CYTB and D-loop haplotype NETWORKs.

| CYTB | | |  | D-loop | | | | | |
| --- | --- | --- | --- | --- | --- | --- | --- | --- | --- |
| Nucleotide position | Number of Mutations | **W** |  | Nucleotide position | Number of Mutations | **W** | Nucleotide position | Number of Mutations | **W** |
| 29 | 4 | **3** |  | 2 | 2 | **9** | 237 | 3 | **9** |
| 47 | 4 | **3** |  | 9 | 2 | **9** | 238 | 4 | **8** |
| 50 | 2 | **7** |  | 19 | 19 | **3** | 240 | 5 | **8** |
| 98 | 2 | **7** |  | 20 | 10 | **6** | 241 | 12 | **6** |
| 102 | 2 | **7** |  | 21 | 8 | **7** | 242 | 3 | **9** |
| 131 | 3 | **5** |  | 22 | 8 | **7** | 247 | 4 | **8** |
| 161 | 2 | **7** |  | 26 | 4 | **8** | 248 | 5 | **8** |
| 188 | 2 | **7** |  | 35 | 2 | **9** | 253 | 7 | **7** |
| 209 | 2 | **7** |  | 79 | 6 | **8** | 257 | 2 | **9** |
| 212 | 2 | **7** |  | 84 | 3 | **9** | 277 | 6 | **8** |
| 230 | 3 | **5** |  | 85 | 8 | **7** | 278 | 3 | **9** |
| 236 | 2 | **7** |  | 86 | 14 | **5** | 282 | 2 | **9** |
| 257 | 2 | **7** |  | 91 | 10 | **6** | 284 | 8 | **7** |
| 278 | 2 | **7** |  | 92 | 10 | **6** | 287 | 2 | **9** |
| 296 | 4 | **3** |  | 93 | 3 | **9** | 293 | 3 | **9** |
| 317 | 3 | **5** |  | 102 | 2 | **9** | 299 | 2 | **9** |
| 359 | 2 | **7** |  | 103 | 2 | **9** | 306 | 3 | **9** |
| 368 | 3 | **5** |  | 107 | 2 | **9** | 309 | 6 | **8** |
| 383 | 2 | **7** |  | 195 | 14 | **5** | 312 | 4 | **8** |
| 434 | 2 | **7** |  | 198 | 7 | **7** | 343 | 8 | **7** |
| 452 | 2 | **7** |  | 207 | 2 | **9** | 354 | 2 | **9** |
| 494 | 4 | **3** |  | 209 | 2 | **9** | 358 | 3 | **9** |
| 512 | 2 | **7** |  | 210 | 3 | **9** | 391 | 2 | **9** |
| 522 | 2 | **7** |  | 212 | 19 | **3** | 400 | 5 | **8** |
| 530 | 2 | **7** |  | 224 | 2 | **9** | 404 | 14 | **5** |
|  |  |  |  | 226 | 3 | **9** | 405 | 3 | **9** |
|  |  |  |  | 227 | 4 | **8** | 406 | 8 | **7** |
|  |  |  |  | 228 | 8 | **7** | 407 | 4 | **8** |
|  |  |  |  | 229 | 9 | **7** | 430 | 5 | **8** |
|  |  |  |  | 230 | 22 | **2** | 477 | 2 | **9** |
|  |  |  |  | 231 | 23 | **2** | 488 | 3 | **9** |
|  |  |  |  | 232 | 11 | **6** | 516 | 3 | **9** |
|  |  |  |  | 233 | 8 | **7** | 517 | 15 | **5** |
|  |  |  |  | 236 | 2 | **9** | 520 | 8 | **7** |

Fig. S5 Extended Bayesian skyline plot derived from the concatenated mtDNA alignment of *E.* gambianus: A) AC and VG populations and B) Ghanaian populations. The $\boldsymbol{x}$ axis is in units of years before 2015, and the $\boldsymbol{y}$ axis is equal to $\boldsymbol{N}\boldsymbol{e}\boldsymbol{\tau}$ (product of the effective population size and the generation time in years). Both axes are in log-scale. The dashed line is the median estimate and the grey area display the 95% highest posterior density.

**A**

**B**

Fig. S6 *Epomophorus gambianus* allele frequency distribution from the colony of Greater Accra. Normal L-shape distribution of alleles grouped in 10 allele frequency classes, obtained from 17 microsatellite loci.

Table S7. Alternative measurements of *F-statistics* between population grouping.

|  |  | EG | Ghana | CT* | rGH | WS | NCG |
| --- | --- | --- | --- | --- | --- | --- | --- |
| *H_O_* | Observed Heterozygosity | 0.82 | 0.85 | 0.85 | 0.85 | 0.83 | 0.69 |
| *H_S_* | Expected Heterozygosity | 0.84 | 0.86 | 0.86 | 0.87 | 0.85 | 0.78 |
| *H_T_* | Total Heterozygosity | 0.87 | 0.87 | 0.87 | 0.87 | 0.86 | 0.84 |
| *H't* | Corrected *H_T_* | 0.87 | 0.87 | 0.87 | 0.87 | 0.87 | NA |
| *G_IS_* | Inbreeding coefficient | 0.04 | 0.02 | 0.02 | 0.02 | 0.03 | 0.12 |
| *G_ST_* | Nei’s fixation index | 0.02 | 0.01 | 0.01 | 0.00 | 0.02 | 0.07 |
| *G'_ST_* | Nei’s corrected *G_ST_* | 0.03 | 0.01 | 0.01 | 0.00 | 0.02 | NA |
| *G'_ST (Hed)_* | Hedrick’s standardised *G_ST_* | 0.17 | 0.05 | 0.05 | 0.01 | 0.14 | 0.48 |
| *G''_ST_* | Corrected *G'_ST (Hed)_* | 0.17 | 0.05 | 0.05 | 0.01 | 0.14 | NA |
| *D_est_* | *Jost’s D* | 0.15 | 0.04 | 0.04 | 0.01 | 0.12 | 0.44 |

CT*: Group with Ghana and CAR populations; rGH: Ghanaian colonies excluding AC and VG; WS: Group with GH, CAR and NG populations; NCE: Group with NG, CAR and ET populations.

|  | Structure tested | % Variance | | *Φ-*statistics | | *p* | % Variance | *F-*statistics | | | *p* |
| --- | --- | --- | --- | --- | --- | --- | --- | --- | --- | --- | --- |
| 1. | **One Group (All populations)** | | | | | |  |  |  |  | |
|  | *Among populations* | | 21.1 |  |  |  | 1.1 |  |  |  | |
|  | *Within populations* | | 78.9 | *Φ_ST_* | 0.21 | **0.00** | 98.9 | *F_ST_* | 0.01 | **0.00** | |
| 2. | **One Group (Ghanaian populations)** | | | | | |  |  |  |  | |
|  | *Among populations* | | 9.4 |  |  |  | 0.8 |  |  |  | |
|  | *Within populations* | | 90.6 | *Φ_ST_* | 0.09 | **0.00** | 99.2 | *F_ST_* | 0.01 | **0.00** | |
| 3. | **One Group (Ghana excluding AC and VG)** | | | | | |  |  |  |  | |
|  | *Among populations* | | 2.5 | *Φ_ST_* | 0.02 | **0.01** | 0.3 |  |  | **0.01** | |
|  | *Within populations* | | 97.5 |  |  |  | 99.7 | *F_ST_* | 0.00 |  | |
| 4. | **One Group (CAR + ET)** | | | | | |  |  |  |  | |
|  | *Among populations* | | 80.1 |  |  |  | 6.18 |  |  |  | |
|  | *Within populations* | | 19.9 | *Φ_ST_* | 0.80 | **0.00** | 93.8 | *F_ST_* | 0.06 | **0.00** | |
| 5. | **Two Groups (Ghana) *vs* (CAR + ET)** | | | | | |  |  |  |  | |
|  | *Among groups* | | *-1.2* | *Φ_CT_* | -0.01 | 0.23 | 1.01 | *F_CT_* | 0.01 | **0.00** | |
|  | *Among populations* | | *21.0* | *Φ_SC_* | 0.20 | **0.00** | 0.95 | *F_SC_* | 0.01 | **0.00** | |
|  | *Within populations* | | *79.6* | *Φ_ST_* | 0.21 | **0.00** | 98.04 | *F_ST_* | 0.02 | **0.00** | |
| 6. | **Two Groups (Ghana + CAR) *vs* (ET)** | | | | | |  |  |  |  | |
|  | *Among groups* | | 78.4 | *Φ_CT_* | 0.78 | 0.08 | 5.54 | *F_CT_* | 0.06 | **0.00** | |
|  | *Among populations* | | 1.9 | *Φ_SC_* | 0.09 | **0.00** | 0.74 | *F_SC_* | 0.01 | **0.00** | |
|  | *Within populations* | | 19.7 | *Φ_ST_* | 0.80 | **0.00** | 93.72 | *F_ST_* | 0.06 | **0.00** | |
| 7. | **Two Groups (AC + VG) *vs* (rGHANA)** | | | | | |  |  |  |  | |
|  | *Among groups* | | 18.1 | *Φ_CT_* | 0.18 | **0.02** | 0.96 | *F_CT_* | 0.01 | **0.02** | |
|  | *Among populations* | | 2.0 | *Φ_SC_* | 0.02 | **0.04** | 0.42 | *F_SC_* | 0.00 | **0.00** | |
|  | *Within populations* | | 79.9 | *Φ_ST_* | 0.20 | **0.00** | 98.62 | *F_ST_* | 0.01 | **0.00** | |
| 9. | **Three Groups (Ghana) *vs* (CAR) *vs* (ET)** | | | | | |  |  |  |  | |
|  | *Among groups* | | 34.1 | *Φ_CT_* | 0.34 | 0.16 | 2.29 | *F_CT_* | 0.02 | **0.00** | |
|  | *Among populations* | | 6.2 | *Φ_SC_* | 0.09 | **0.00** | 1.75 | *F_SC_* | 0.01 | **0.00** | |
|  | *Within populations* | | 59.7 | *Φ_ST_* | 0.40 | **0.00** | 96.96 | *F_ST_* | 0.03 | **0.00** | |
| 10. | **Four Groups (AC + VG) *vs* (rGHANA) *vs* (CAR) *vs* (ET)** | | | | | | | | | | |
|  | *Among groups* | | 30.6 | *Φ_CT_* | 0.31 | **0.01** | 1.5 | *F_CT_* | 0.02 | | **0.00** |
|  | *Among populations* | | 1.7 | *Φ_SC_* | 0.02 | 0.02 | 0.43 | *F_SC_* | 0.00 | | **0.00** |
|  | *Within populations* | | 67.7 | *Φ_ST_* | 0.32 | **0.00** | 98.07 | *F_ST_* | 0.02 | | **0.00** |

**Table S8. Hierarchical AMOVA analysis and population structure using: mtDNA CYTB (*Φ-*statistics) and ncDNA microsatellites (*F-*statistics). *P* values (*p*) below 0.05 are noted in bold. rGHANA: Ghanaian colonies excluding AC and VG. Analyses of ET produced only with SI colony.**

Fig. S9. Analyses of isolation using pairwise comparisons between logged geographical distances and genetic distances. Genetic distances of mtDNA-CYTB (*Φ_ST_* (*Φ_ST_/1- Φ_ST_*) and ncDNA- microsatellites (*F_ST_* (*F_ST_/1- F_ST_*). Analyses were performed using Mantel tests with 10,000 iterations; *p* values and adjusted *R^2^* values are shown in the plots (the scales vary between plots).


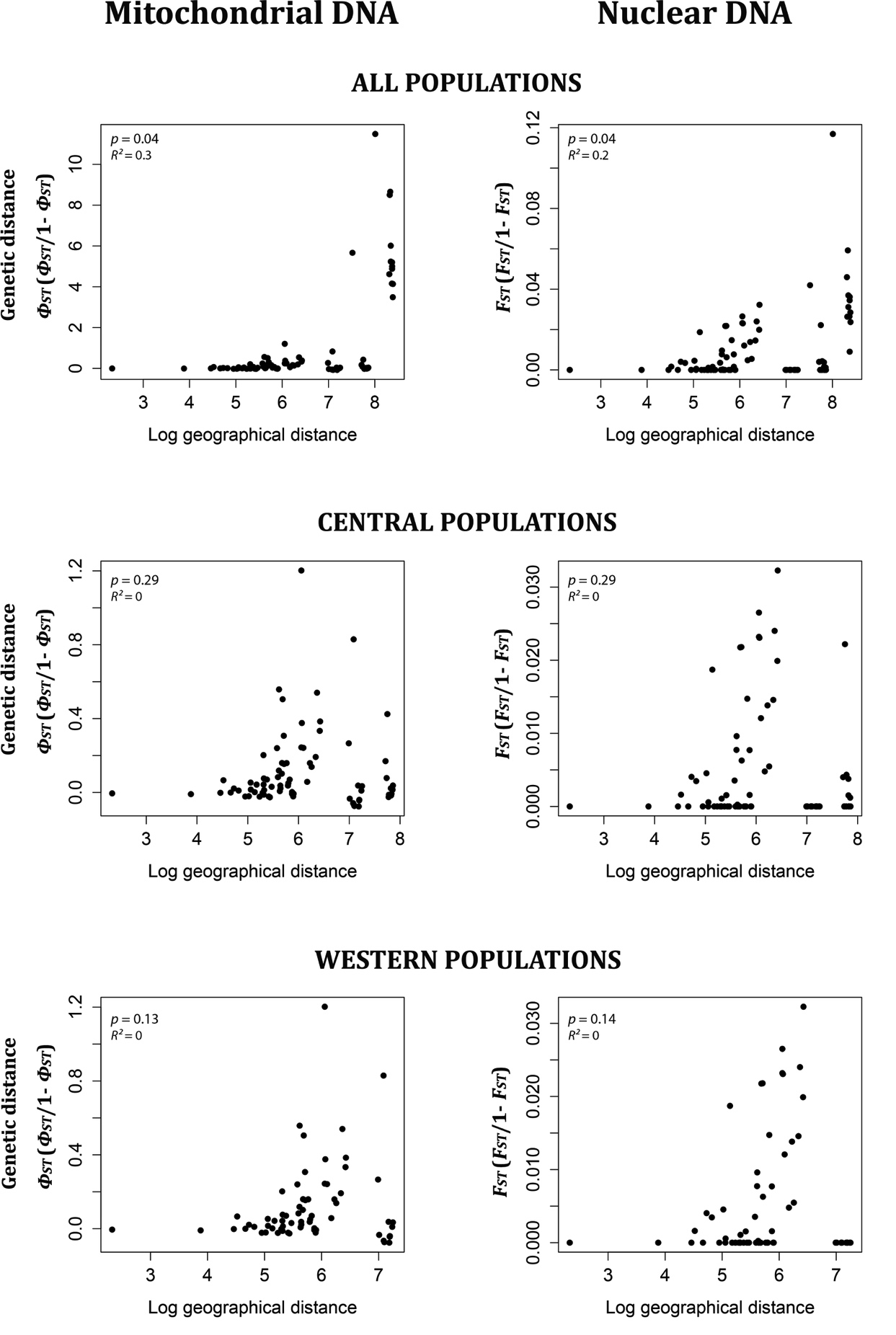


**H**

**C**

**G**

**B**

**A**

**F**

Fig. S9. Continuation.


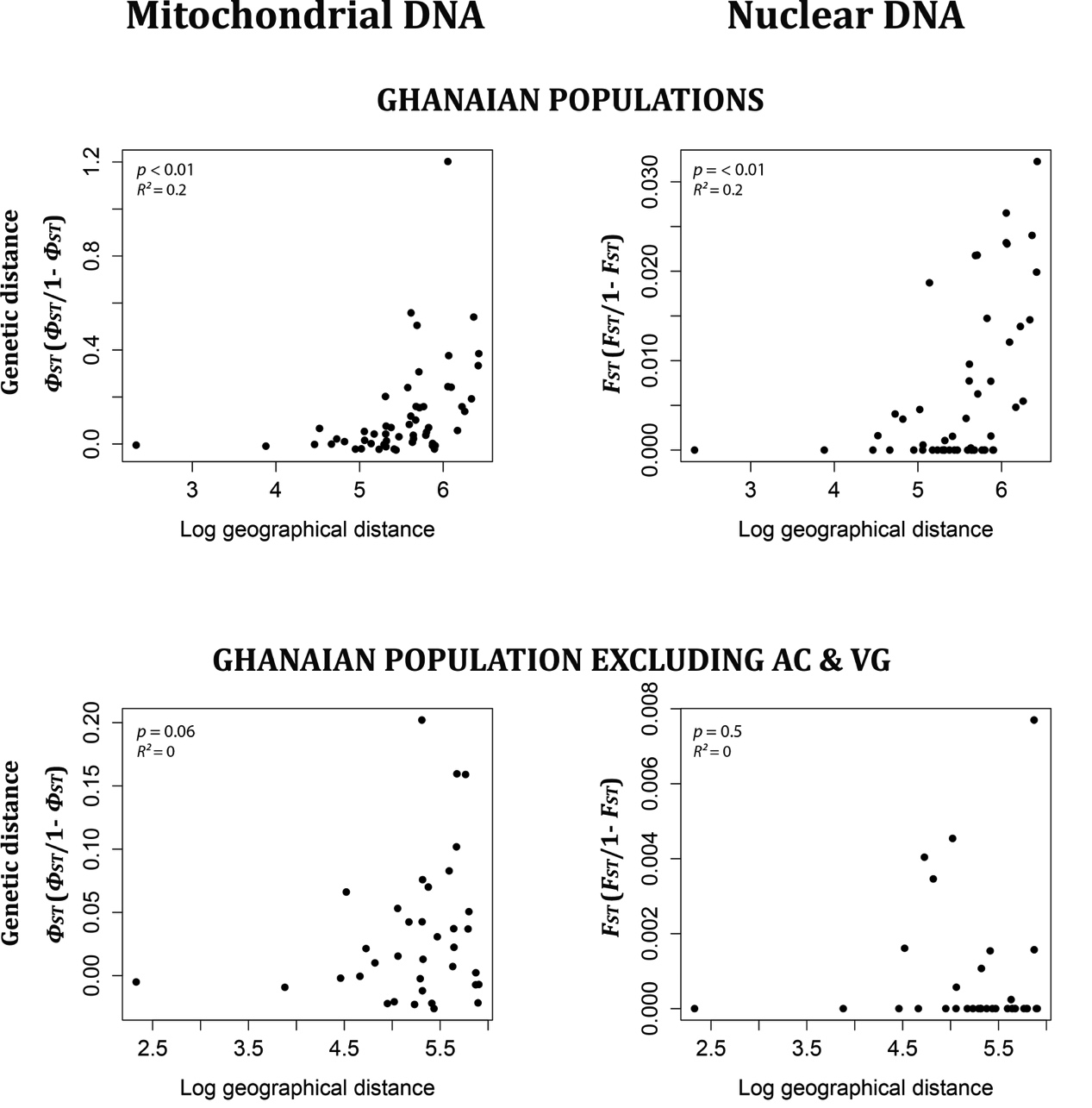


**D**

**I**

**J**

**E**

Fig. S10. Population genetic Bayesian clustering with the software STRUCTURE. An Admixture model using population information as prior was run with 277 bats. Genetic clusters (K) are visualised as a colour. Each individual is represented as a vertical line, coloured proportionally to the membership assignment of K.


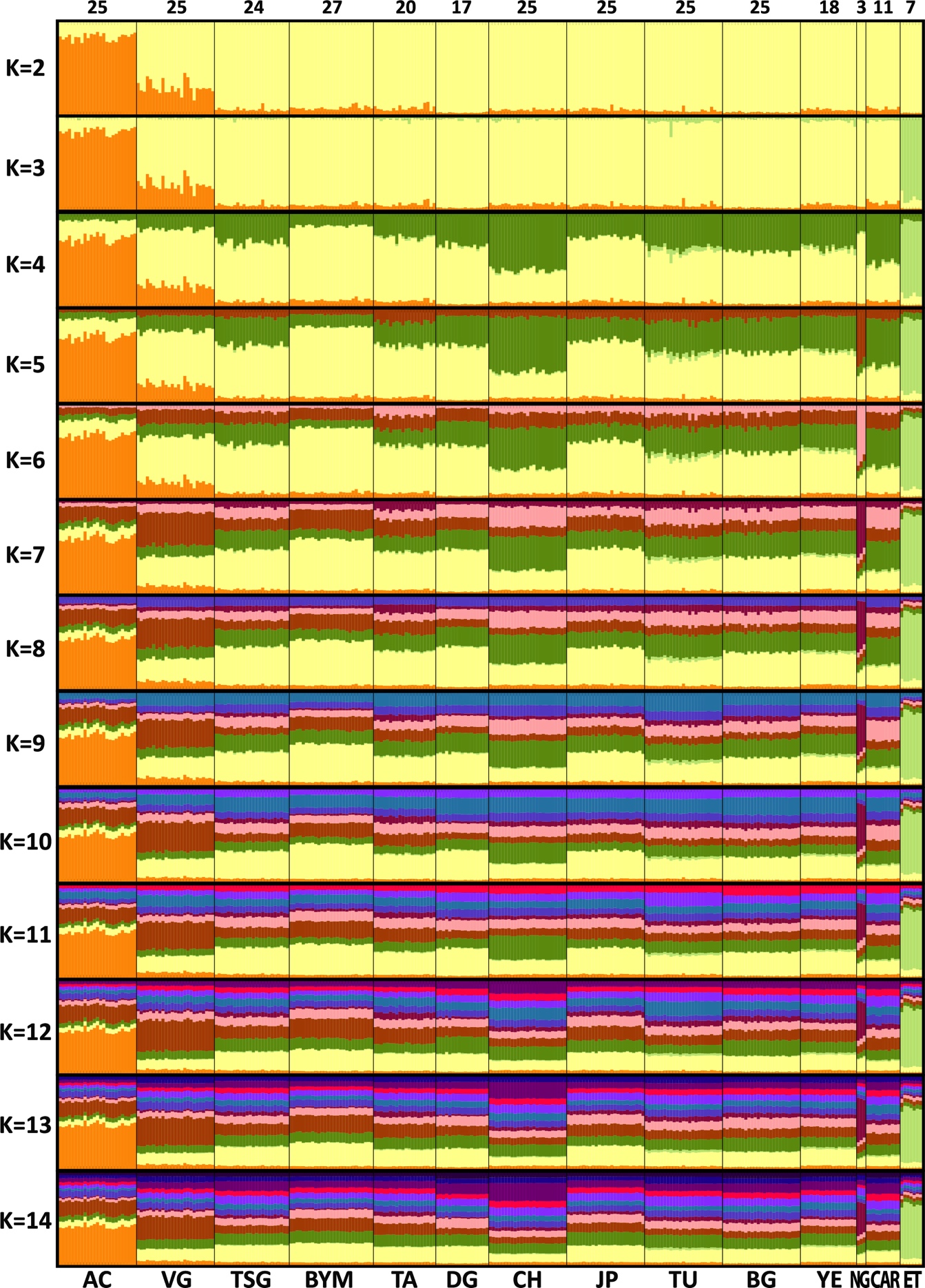


Fig. S11. Delta K optimal clustering of two STRUCTURE models. Estimation of the optimal genetic cluster using Delta K, which is based on the rate of change in the log probability of data between successive *K* values, K=4 was identified as the best fit number of genetic divisions for both models. A: STRUCTURE Admixture model run with 15 microsatellite loci in 277 individuals using location information as prior. B: Same parameters as A, done with 249 individuals (to allow averaged sample numbers).


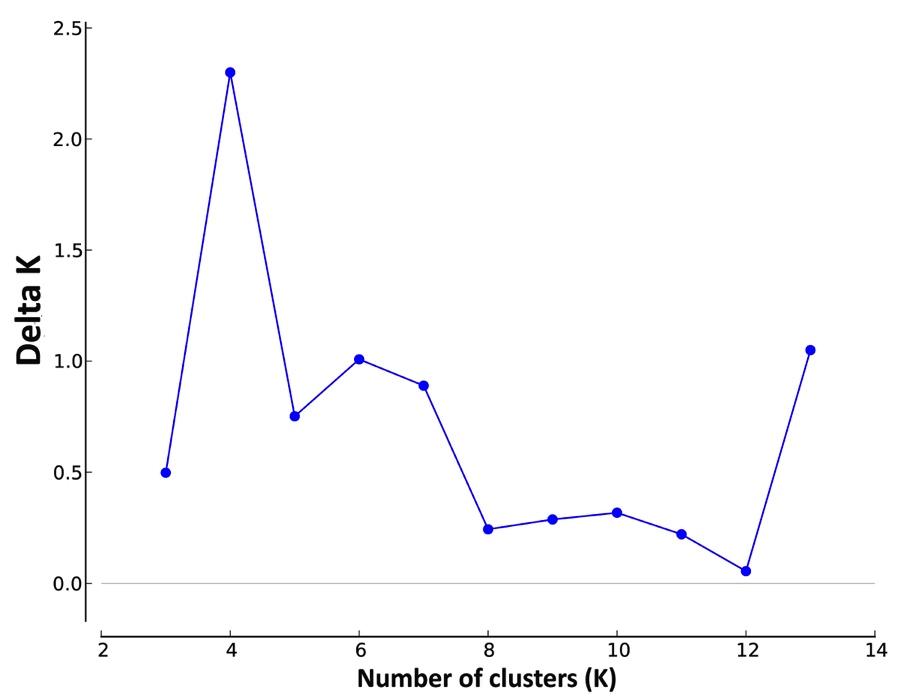


**A**


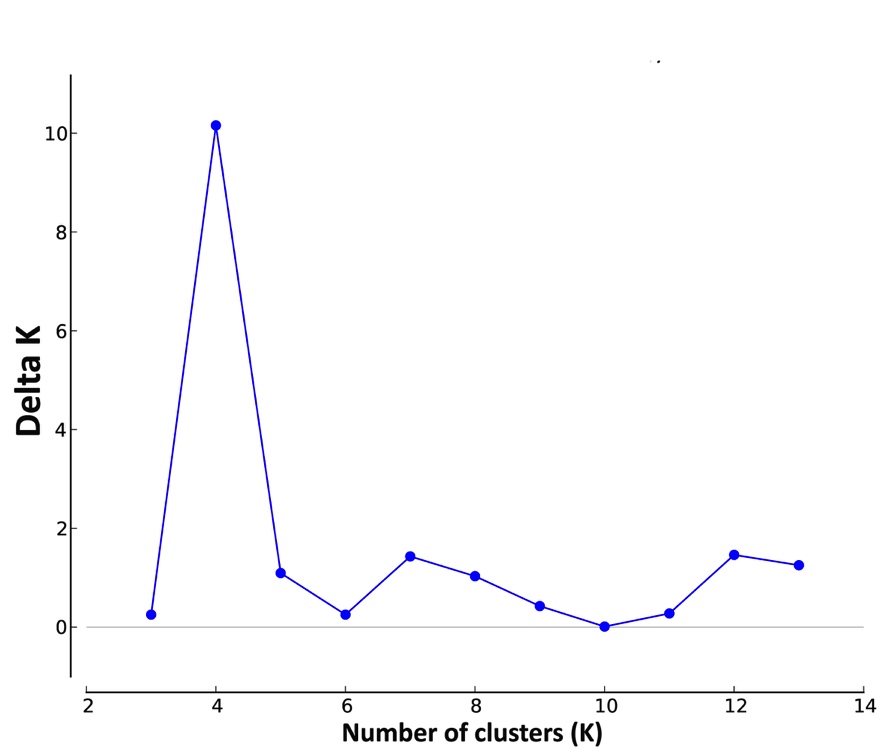


**B**

Fig. S12. Weighted haplotype networks of *E. gambianus*. A) CYTB median joining haplotype networks at the country level. B) D-loop median joining haplotype networks at the colony level. Circle size is proportional to the frequency of specimens sharing that haplotype and the colour reflects the population of origin. The lines between two haplotypes show base substitutions, and its length is proportional to the number of point mutations. There is a consistent spatial clustering, between the Ethiopian colonies (in black) and the rest of the African populations, using both mitochondrial markers.


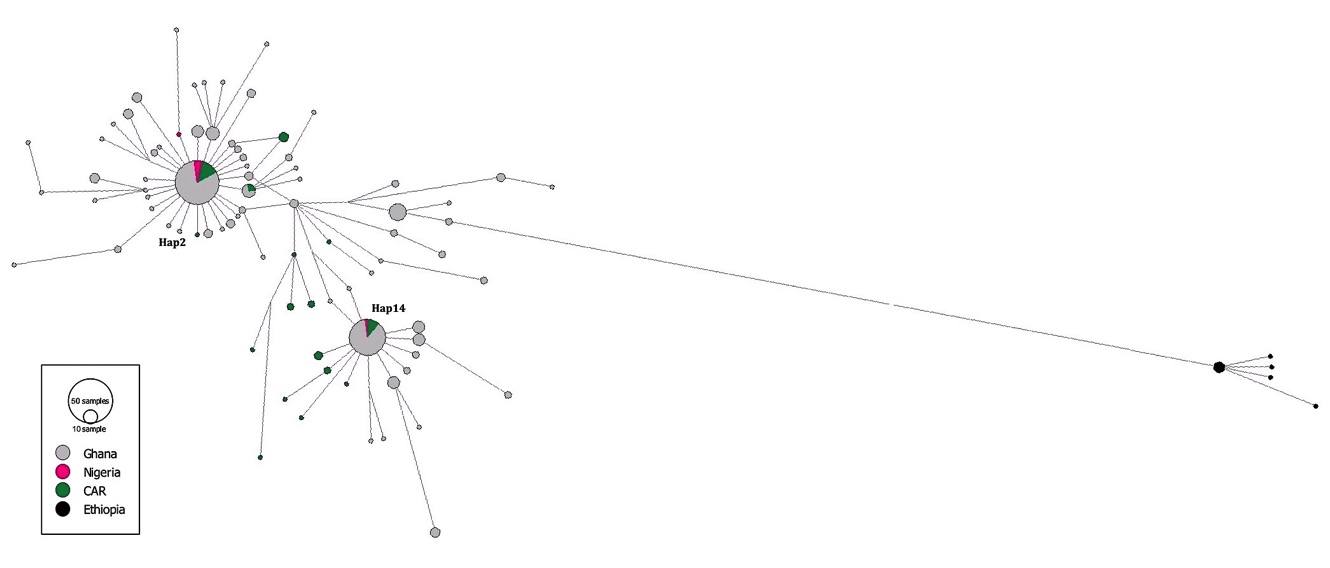


**A**


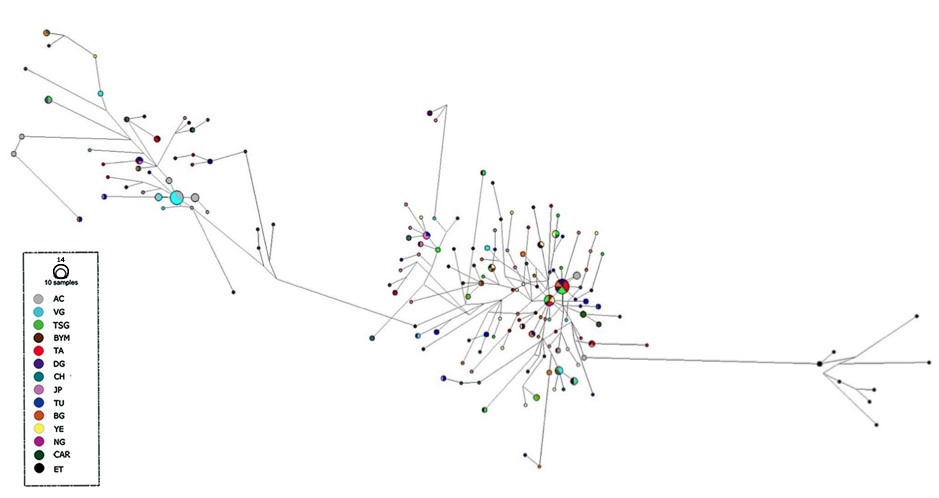


**B**

Fig. S13A. Phylogeny of *E. gambianus* concatenated CYTB and D-loop fragments. Bayesian phylogenetic tree using a HKY+I+G substitution model and a 937 bp alignment. The Ethiopian clade is noted in bold and with a black line. Hap 2 and 111 are typed in blue. Outgroup species are typed in red. *Rousettus aegyptiacus is indicated with an asterisk*.


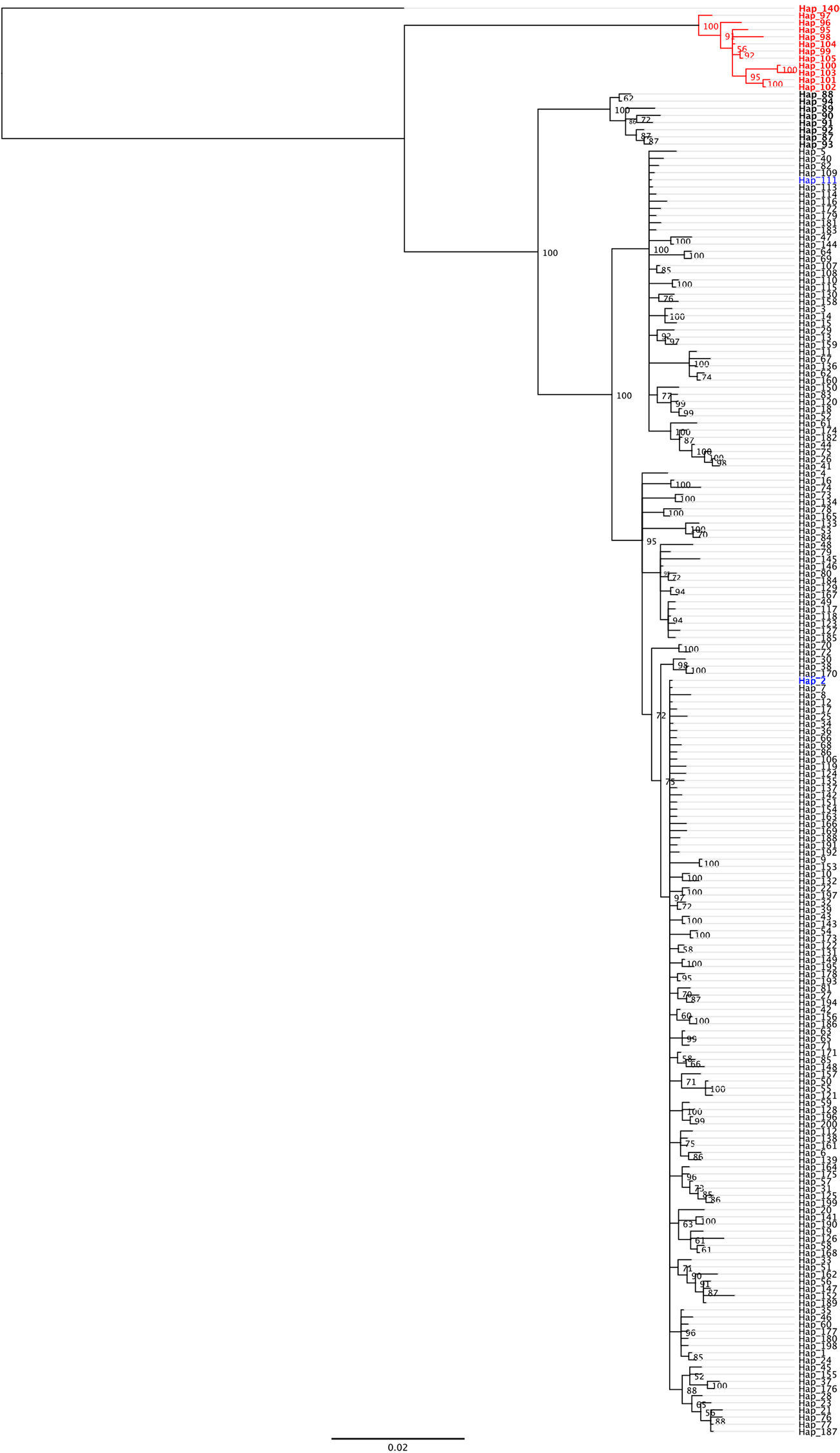


*


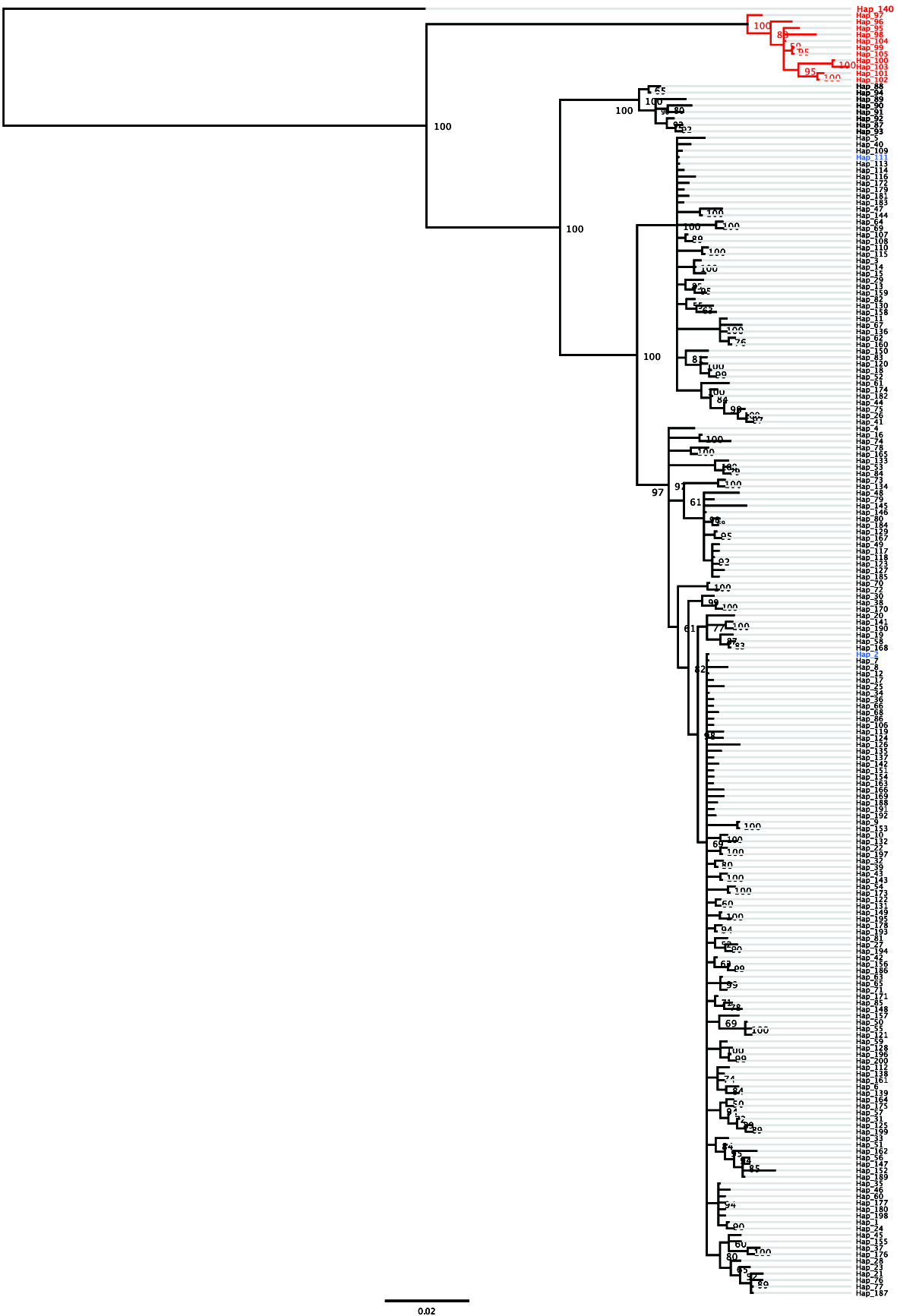
Fig. S13B. Phylogeny of *E. gambianus* concatenated CYTB and D-loop fragments. Bayesian phylogenetic tree using a mixed gamma substitution model and a 937 bp alignment. The Ethiopian clade is noted in bold and with a black line. Hap 2 and 111 are typed in blue. Outgroup species are typed in red. *Rousettus aegyptiacus is indicated with an asterisk*.

*

**Fig. S14.** **Map showing *E. gambianus* geographical distribution (extracted from IUCN and shown with an orange line) and sampling sites (circles). The legend shows the colour coded sampling sites, where AC: Greater Accra, VG: Ve-Golokwati, TSG: Tanoboase Sacred Grove, BYM: Buoyem, TA: Tamale, DG: Damongo, CH: Charia, JP: Jirapa, TU: Tumu, BG: Bolgatanga, YE: Yendi, CAR: Central African Republic, NG: Nigeria, ET: Ethiopia, CAR^†^: CYTB sequences downloaded from GenbBank. Bubble size reflects sample size and darker colour denotes increasing forest loss. Layers retrieved from ESRI, where “tree cover” is defined as all vegetation greater than 5 meters in height (map above) or 3 meters (bottom map); “Loss” indicates the removal or mortality of tree cover. Data obtained during 2011-2014 (map above) and 2013-2014 (bottom map).**

**
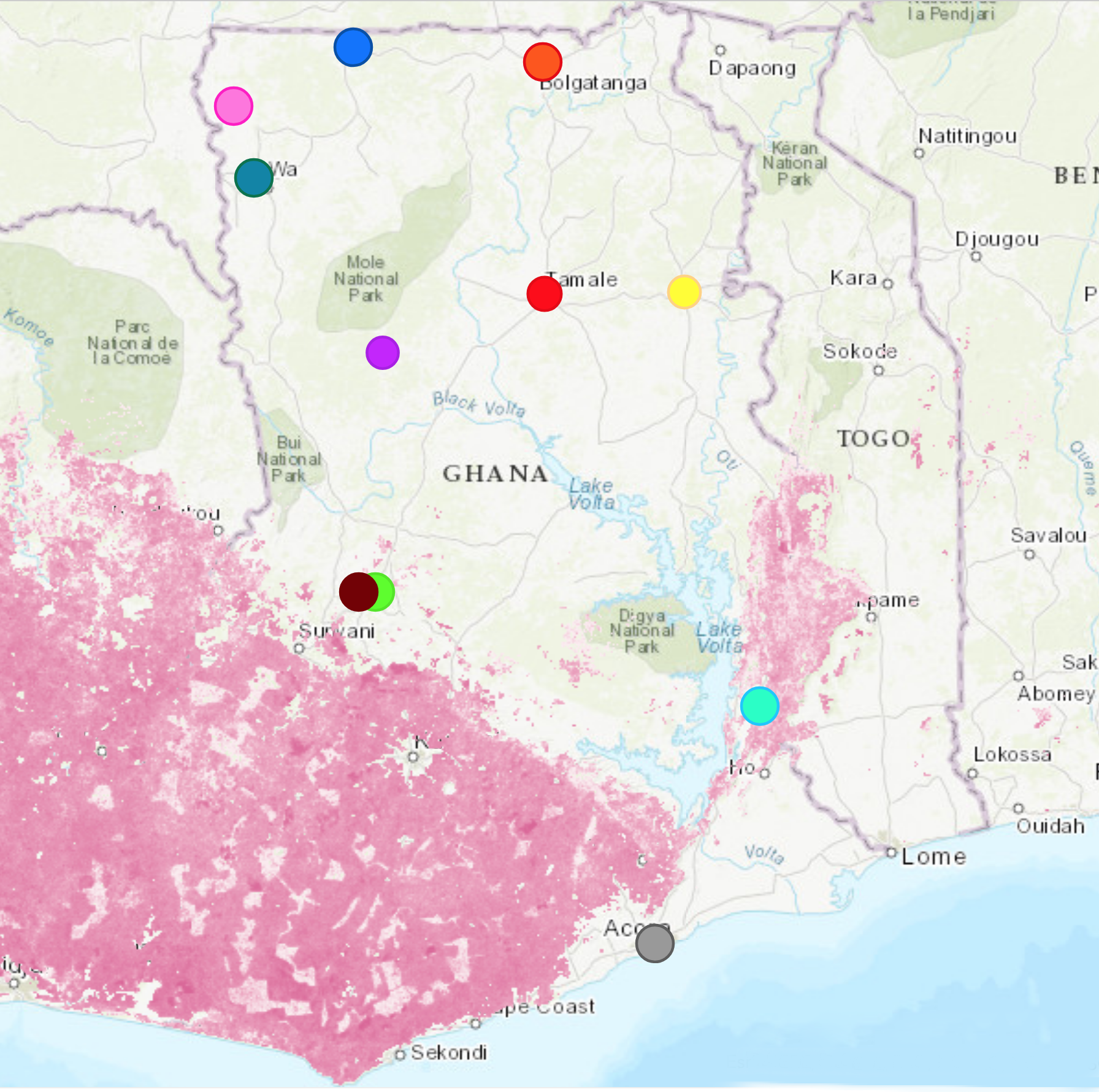

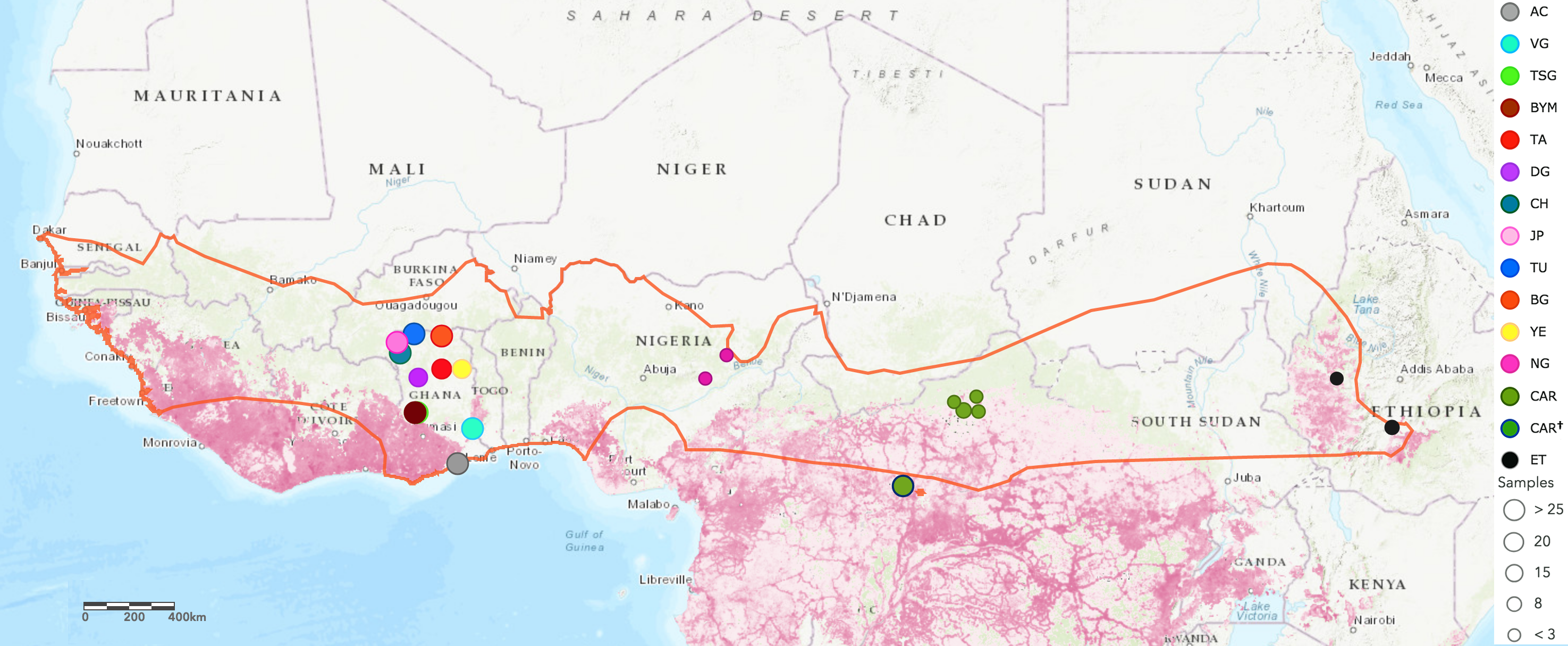
**

**
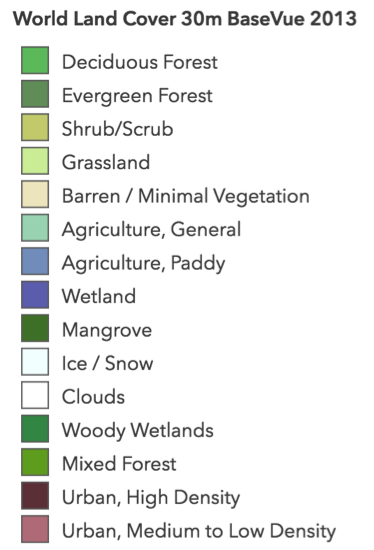
**

**
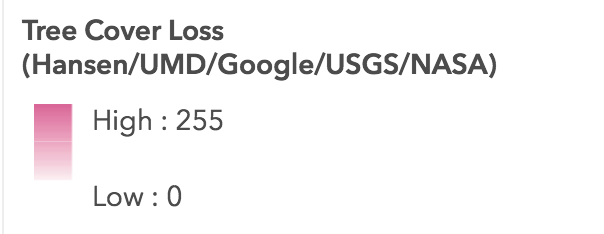
**

**
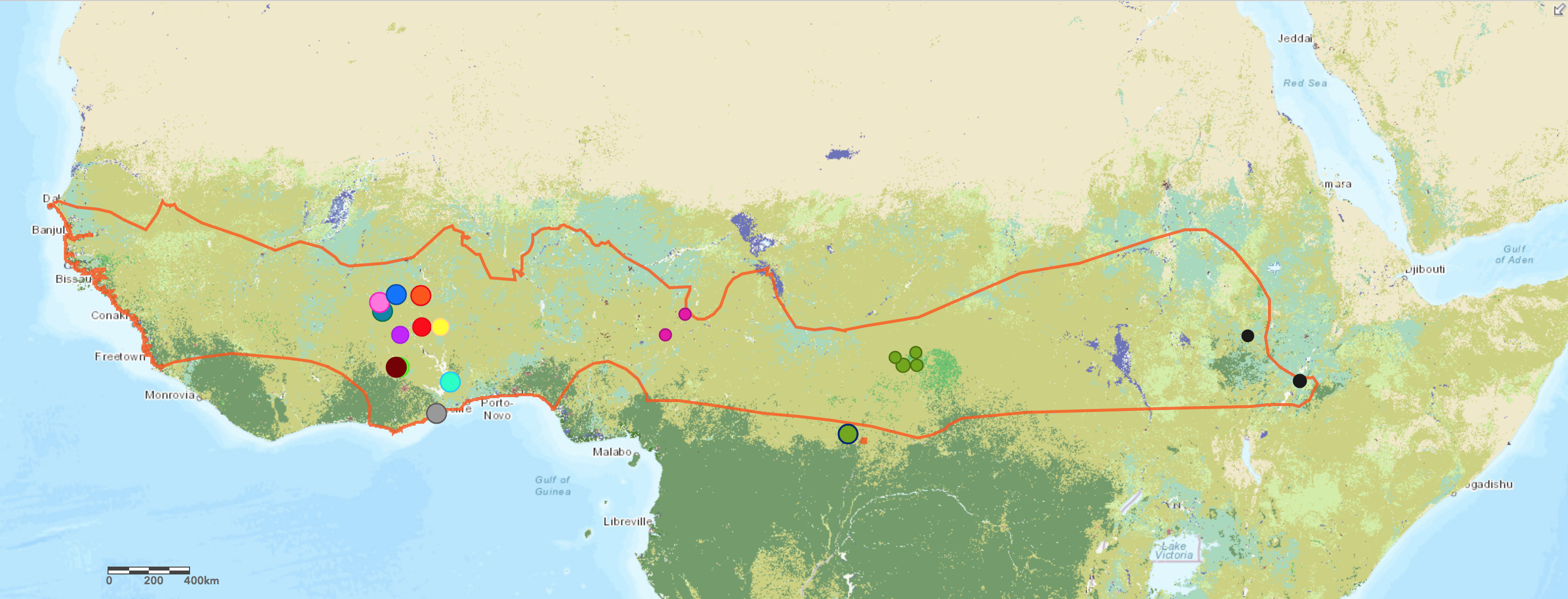
**

**Fig. S15.** **Geographic map of *E. gambianus* haplotype distributions using: A) CYTB *haplotype alignment; B) D-loop haplotype alignment*. Circle size is proportional to bat sampled in each colony and the colour reflects each haplotype. Bright colours were used to highlight haplotypes that were shared in at least two colonies.**


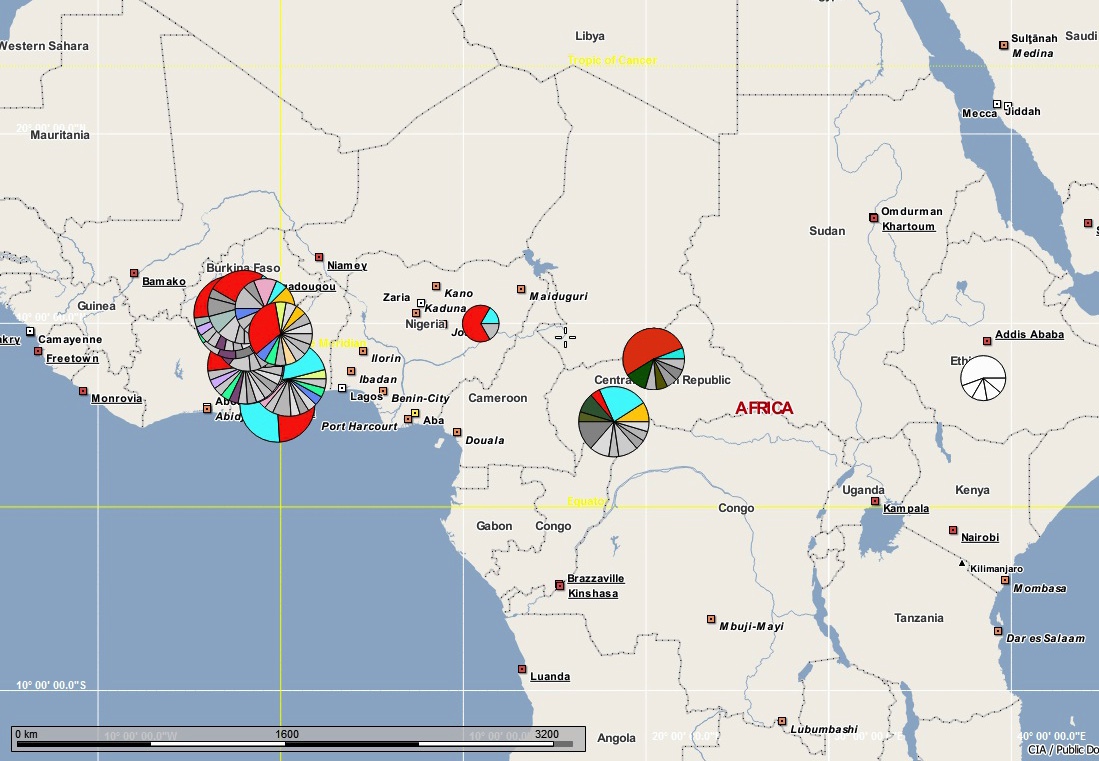

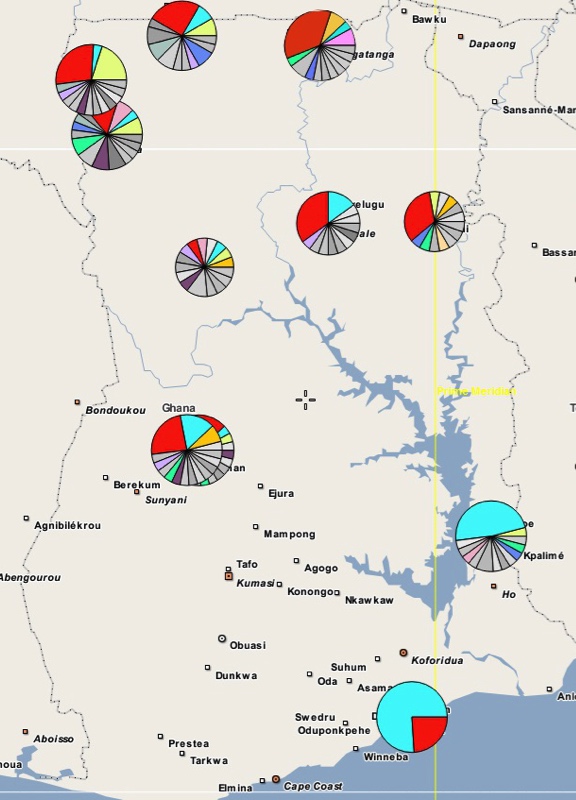


**A *haplotype***


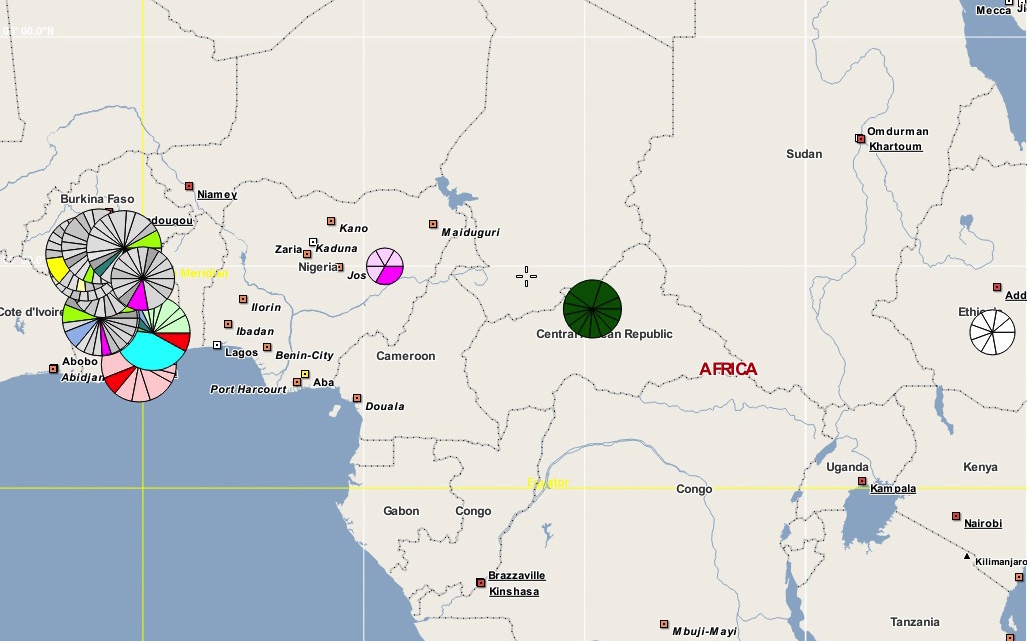

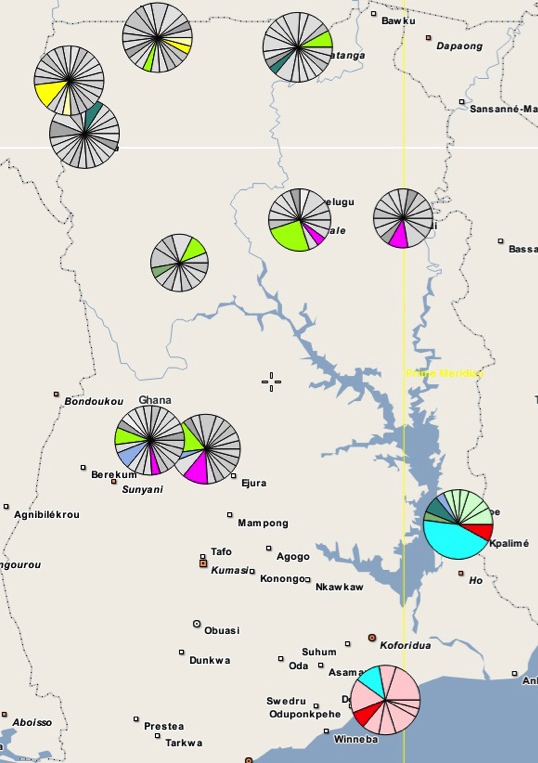


**B *haplotype***
